# Supplementary material for: Sensory sharpening and semantic prediction errors unify competing models of predictive processing in human speech comprehension
Source: PLoS Biol. 2026 Jan 9;24(1):e3003588. doi: 10.1371/journal.pbio.3003588 (PMC12788694; doi:10.1371/journal.pbio.3003588)
Supplement: S1 Table — A generalised linear model revealed that participants reported hearing words as a function of their probability given the speaker prior (fit), remaining perceptual differences (κ), and trial number (t). (PDF) [file pbio.3003588.s014.pdf]

Coefficient	Estimate	Std. Error	z-value	p-value
(Intercept)	0.27	0.06	4.42	9.824392e-06
t	-0.04	0.03	-1.47	1.413237e-01
κ	-0.62	0.11	-5.81	6.261726e-09
fit	1.64	0.12	13.11	2.712021e-39
$t \times \kappa$	0.39	0.06	6.09	1.135522e-09
$t\times$fit	0.43	0.03	12.77	2.405336e-37

S1 Table. Behavioural analysis in online experiment. A generalised linear model revealed that participants reported hearing words as a function of their probability given the speaker prior (*fit*), remaining perceptual differences (κ), and trial number (t).
