# Supplementary material for: Sensory sharpening and semantic prediction errors unify competing models of predictive processing in human speech comprehension
Source: PLoS Biol. 2026 Jan 9;24(1):e3003588. doi: 10.1371/journal.pbio.3003588 (PMC12788694; doi:10.1371/journal.pbio.3003588)
Supplement: S2 Table — Effects of the probability of a word given the speaker prior (fit), remaining perceptual differences (κ), and trial number (t) on the reported word were replicated in the EEG experiment. (PDF) [file pbio.3003588.s015.pdf]

Coefficient	Estimate	Std. Error	z-value	p-value
(Intercept)	0.13	0.05	2.84	4.546977e-03
t	-0.26	0.03	-8.00	1.197850e-15
κ	-0.14	0.09	-1.60	1.102186e-01
fit	1.35	0.06	20.99	8.269810e-98
$t \times \kappa$	0.42	0.06	6.62	3.606962e-11
$t\times$ fit	0.38	0.03	11.07	1.811167e-28

S2 Table. Replication of behavioural analysis in EEG experiment. Effects of the probability of a word given the speaker prior (*fit*), remaining perceptual differences (κ), and trial number (t) on the reported word were replicated in the EEG experiment.
