# Supplementary material for: Sensory sharpening and semantic prediction errors unify competing models of predictive processing in human speech comprehension
Source: PLoS Biol. 2026 Jan 9;24(1):e3003588. doi: 10.1371/journal.pbio.3003588 (PMC12788694; doi:10.1371/journal.pbio.3003588)
Supplement: S3 Table — Across all 28 frequency bands of the gammatone filterbank, stimulus reconstruction performance was significantly above chance level. (PDF) [file pbio.3003588.s016.pdf]

Frequency band	M	Std. Dev.	df	t-value	p-value
0	0.06	0.03	34	10.56	3.151979e-11
1	0.06	0.03	34	11.03	1.352901e-11
2	0.06	0.04	34	9.30	5.005196e-10
3	0.06	0.04	34	9.45	3.847424e-10
4	0.06	0.04	34	10.13	7.584404e-11
5	0.07	0.03	34	12.00	1.983854e-12
6	0.08	0.03	34	13.22	1.423830e-13
7	0.08	0.04	34	12.86	2.992608e-13
8	0.09	0.04	34	14.33	1.534314e-14
9	0.09	0.04	34	13.60	6.844391e-14
10	0.08	0.04	34	11.84	2.616005e-12
11	0.07	0.04	34	10.90	1.581847e-11
12	0.06	0.03	34	10.69	2.490932e-11
13	0.06	0.03	34	11.21	9.356256e-12
14	0.07	0.03	34	11.35	7.484068e-12
15	0.07	0.04	34	11.34	7.129978e-12
16	0.06	0.03	34	11.00	1.357066e-11
17	0.07	0.03	34	11.36	7.745446e-12
18	0.06	0.03	34	11.99	1.924524e-12
19	0.05	0.03	34	10.25	6.210367e-11
20	0.03	0.02	34	7.44	6.303351e-08
21	0.03	0.03	34	5.87	2.529152e-06
22	0.03	0.03	34	6.01	2.514777e-06
23	0.03	0.03	34	5.74	1.852334e-06
24	0.03	0.03	34	6.87	2.638222e-07
25	0.04	0.02	34	8.32	6.177527e-09
26	0.06	0.02	34	13.36	1.084790e-13
27	0.10	0.04	34	15.24	2.594835e-15

S3 Table. Stimulus reconstruction per frequency band. Across all 28 frequency bands of the gammatone filterbank, stimulus reconstruction performance was significantly above chance level.
