# Supplementary material for: Sensory sharpening and semantic prediction errors unify competing models of predictive processing in human speech comprehension
Source: PLoS Biol. 2026 Jan 9;24(1):e3003588. doi: 10.1371/journal.pbio.3003588 (PMC12788694; doi:10.1371/journal.pbio.3003588)
Supplement: S4 Table — Results from consecutive contrasts of top–k within-item cRSM regression models showed consistent increases in model performance, albeit with diminishing returns for larger k. (PDF) [file pbio.3003588.s017.pdf]

contrast	M	Std. Dev	df	t-value	p-value
3-1	0.092	0.011	34	49.284	2.954380e-32
5-3	0.034	0.007	34	27.689	5.264768e-24
7-5	0.014	0.003	34	24.428	2.674880e-22
9-7	0.008	0.004	34	13.033	5.308751e-14
11-9	0.004	0.003	34	9.122	5.810556e-10
13-11	0.004	0.003	34	8.287	4.540814e-09
15-13	0.003	0.002	34	7.560	2.655277e-08
17-15	0.001	0.002	34	3.150	3.398163e-03
19-17	0.003	0.002	34	6.885	1.250519e-07

S4 Table. Influence of the number of predicted words on cRSA regression performance. Results from consecutive contrasts of top$-k$ within-item cRSM regression models showed consistent increases in model performance, albeit with diminishing returns for larger k.
