# Supplementary material for: Sensory sharpening and semantic prediction errors unify competing models of predictive processing in human speech comprehension
Source: PLoS Biol. 2026 Jan 9;24(1):e3003588. doi: 10.1371/journal.pbio.3003588 (PMC12788694; doi:10.1371/journal.pbio.3003588)
Supplement: S5 Table — Results from contrasts in within-item cRSM regression models using k = 19 confirmed the pattern reported for k = 5. (PDF) [file pbio.3003588.s018.pdf]

contrast	M	Std. Dev	df	t-value	p-value
ac.inv.-baseline	0.0032	0.0014	34	13.1557	1.083377e-13
ac.spc.-baseline	0.0066	0.0013	34	29.3987	2.415507e-24
ac.spc.-ac.inv.	0.0034	0.0014	34	14.1754	1.331002e-14
ac.bth.-baseline	0.0107	0.0017	34	36.9431	1.487629e-27
ac.bth.-ac.inv.	0.0075	0.0015	34	29.7752	1.714360e-24
ac.bth.-ac.spc.	0.0041	0.0011	34	20.7433	1.481206e-19
sem.inv.-baseline	-0.0001	0.0004	34	-0.8850	1.000000e+00
sem.spc.-baseline	-0.0004	0.0002	34	-8.3291	1.312180e-08
sem.spc.-sem.inv.	-0.0003	0.0004	34	-4.4158	8.721333e-04
sem.bth.-baseline	-0.0005	0.0004	34	-6.8839	7.533303e-07
sem.bth.-sem.inv.	-0.0005	0.0001	34	-22.5252	1.133089e-20
sem.bth.-sem.spc.	-0.0002	0.0004	34	-2.5786	1.153901e-01
sem.inv.-ac.inv.	-0.0031	0.0032	34	-5.6651	2.346169e-05
sem.spc.-ac.spc.	-0.0068	0.0026	34	-15.5283	9.511598e-16
sem.bth.-ac.bth.	-0.0111	0.0028	34	-23.0672	5.779476e-21

S5 Table. cRSM regressions using top-19 predictions. Results from contrasts in within-item cRSM regression models using $k = 19$ confirmed the pattern reported for $k = 5$.
