# Supplementary material for: Sensory sharpening and semantic prediction errors unify competing models of predictive processing in human speech comprehension
Source: PLoS Biol. 2026 Jan 9;24(1):e3003588. doi: 10.1371/journal.pbio.3003588 (PMC12788694; doi:10.1371/journal.pbio.3003588)
Supplement: S6 Table — Comparing cRSM regressions using top-k predictions derived from participant-specific and plausible foil priors showed better performance of participant-specific priors (see Validating top-k acoustic predictions). This indicates that they captured meaningful acoustic predictions by participants. (PDF) [file pbio.3003588.s019.pdf]

contrast	M	Std. Dev	df	t-value	p-value
ac.inv.: true-foil	0.002073	0.007127	34	1.696327	3.958520e-01
ac.spc: true-foil	0.007507	0.006593	34	6.638746	7.745975e-07
ac.both: true-foil	0.007945	0.007985	34	5.801883	7.787670e-06
sem.inv.: true-foil	-0.000825	0.006090	34	-0.790213	1.000000e+00
sem.spc: true-foil	-0.000710	0.006138	34	-0.674545	5.045258e-01
sem.both: true-foil	-0.000769	0.006059	34	-0.739759	9.290471e-01

S6 Table. Top-k predictions capture meaningful acoustic expectations. Comparing cRSM regressions using top-k predictions derived from participant-specific and plausible foil priors showed better performance of participant-specific priors (see Validating top-k acoustic predictions). This indicates that they captured meaningful acoustic predictions by participants.
