# Supplementary material for: Sensory sharpening and semantic prediction errors unify competing models of predictive processing in human speech comprehension
Source: PLoS Biol. 2026 Jan 9;24(1):e3003588. doi: 10.1371/journal.pbio.3003588 (PMC12788694; doi:10.1371/journal.pbio.3003588)
Supplement: S7 Table — Results from between-item RSA regression models using k = 1 (see Conventional representational similarity analysis) mirrored results obtained from cRSA regressions. (PDF) [file pbio.3003588.s020.pdf]

contrast	M	Std. Dev	df	t-value	p-value
baseline-acc.inv. (sh)	-0.008	0.004	34	-12.56	6.8e-13
baseline-acc.spc. (sh)	-0.010	0.005	34	-12.14	1.6e-12
baseline-acc.bth. (sh)	-0.017	0.006	34	-17.09	1.2e-16
baseline-acc.inv. (pe)	-0.005	0.002	34	-12.85	3.9e-13
baseline-acc.spc. (pe)	-0.004	0.002	34	-14.34	2.0e-14
baseline-acc.bth. (pe)	-0.009	0.003	34	-20.08	8.6e-19
baseline-acc.bth. (sh+pe)	-0.024	0.006	34	-24.36	2.1e-21
baseline-sem.inv. (sh)	-0.006	0.003	34	-13.01	2.9e-13
baseline-sem.spc. (sh)	-0.007	0.005	34	-8.10	3.0e-08
baseline-sem.bth. (sh)	-0.012	0.005	34	-13.95	4.2e-14
baseline-sem.inv. (pe)	-0.010	0.005	34	-12.24	1.3e-12
baseline-sem.spc. (pe)	-0.002	0.002	34	-5.63	2.6e-05
baseline-sem.bth. (pe)	-0.012	0.005	34	-12.86	3.9e-13
baseline-sem.bth. (sh+pe)	-0.022	0.007	34	-17.05	1.3e-16
baseline-acc.sem. (sh)	-0.027	0.007	34	-23.14	1.1e-20
baseline-acc.sem. (pe)	-0.019	0.005	34	-21.90	5.8e-20
baseline-acc.sem. (sh+pe)	-0.041	0.008	34	-29.33	5.1e-24
acc.inv. (sh)-acc.spc. (sh)	-0.002	0.006	34	-2.08	2.2e-01
acc.inv. (sh)-acc.bth. (sh)	-0.009	0.004	34	-11.49	6.2e-12
acc.spc. (sh)-acc.bth. (sh)	-0.007	0.003	34	-12.04	1.9e-12
acc.inv. (pe)-acc.spc. (pe)	0.000	0.003	34	0.55	5.9e-01
acc.inv. (pe)-acc.bth. (pe)	-0.004	0.002	34	-13.64	7.9e-14
acc.spc. (pe)-acc.bth. (pe)	-0.004	0.002	34	-11.90	2.5e-12
acc.inv. (sh)-acc.inv. (pe)	0.003	0.005	34	3.71	5.9e-03
acc.spc. (sh)-acc.spc. (pe)	0.005	0.004	34	7.04	5.6e-07
acc.bth. (sh)-acc.bth. (pe)	0.008	0.006	34	7.46	1.8e-07
acc.bth. (sh)-acc.bth. (sh+pe)	-0.007	0.002	34	-20.06	8.7e-19
acc.bth. (pe)-acc.bth. (sh+pe)	-0.015	0.005	34	-16.47	3.5e-16
sem.inv. (sh)-sem.spc. (sh)	-0.001	0.005	34	-1.43	4.9e-01
sem.inv. (sh)-sem.bth. (sh)	-0.007	0.004	34	-8.81	4.6e-09
sem.spc. (sh)-sem.bth. (sh)	-0.005	0.003	34	-10.93	2.2e-11
sem.inv. (pe)-sem.spc. (pe)	0.008	0.004	34	10.71	3.5e-11
sem.inv. (pe)-sem.bth. (pe)	-0.002	0.002	34	-6.97	6.3e-07
sem.spc. (pe)-sem.bth. (pe)	-0.010	0.005	34	-11.39	7.6e-12
sem.inv. (sh)-sem.inv. (pe)	-0.004	0.005	34	-4.73	3.4e-04
sem.spc. (sh)-sem.spc. (pe)	0.005	0.005	34	5.99	9.6e-06
sem.bth. (sh)-sem.bth. (pe)	0.001	0.006	34	0.85	8.1e-01
sem.bth. (sh)-sem.bth. (sh+pe)	-0.009	0.004	34	-12.10	1.7e-12
sem.bth. (pe)-sem.bth. (sh+pe)	-0.010	0.004	34	-14.14	3.0e-14
acc.bth. (sh)-sem.bth. (sh)	0.004	0.008	34	3.23	1.9e-02
acc.bth. (pe)-sem.bth. (pe)	-0.003	0.006	34	-2.62	7.7e-02

contrast	M	Std. Dev	df	t-value	p-value
acc.bth. (sh+pe)-sem.bth. (sh+pe)	0.002	0.009	34	1.45	6.3e-01
acc.bth. (sh)-acc.sem. (sh)	-0.010	0.004	34	-13.37	1.4e-13
sem.bth. (sh)-acc.sem. (sh)	-0.014	0.005	34	-16.29	4.8e-16
acc.bth. (pe)-acc.sem. (pe)	-0.010	0.005	34	-12.70	5.1e-13
sem.bth. (pe)-acc.sem. (pe)	-0.008	0.002	34	-18.21	1.7e-17
acc.sem. (sh)-acc.sem. (pe)	0.007	0.006	34	6.75	1.1e-06
acc.sem. (sh)-acc.sem. (sh+pe)	-0.014	0.004	34	-21.38	1.2e-19
acc.sem. (pe)-acc.sem. (sh+pe)	-0.021	0.005	34	-23.88	3.9e-21
acc.bth. (sh+pe)-acc.sem. (sh+pe)	-0.017	0.006	34	-15.63	1.6e-15
sem.bth. (sh+pe)-acc.sem. (sh+pe)	-0.019	0.005	34	-23.07	1.1e-20

S7 Table. Conventional RSA corroborates findings from cRSA. Results from between-item RSA regression models using $k = 1$ (see Conventional representational similarity analysis) mirrored results obtained from cRSA regressions.
