# Supplementary material for: Sensory sharpening and semantic prediction errors unify competing models of predictive processing in human speech comprehension
Source: PLoS Biol. 2026 Jan 9;24(1):e3003588. doi: 10.1371/journal.pbio.3003588 (PMC12788694; doi:10.1371/journal.pbio.3003588)
Supplement: S8 Table — Results from between-item RSA regression models using k = 1 and restricting the temporal window to 0.0-0.48s to rule out prediction length as a confounding factor (see Conventional representational similarity analysis). (PDF) [file pbio.3003588.s021.pdf]

contrast	M	Std. Dev	df	t-value	p-value
baseline-acc.inv. (sh)	-0.008	0.004	34	-11.22	1.7e-11
baseline-acc.spc. (sh)	-0.010	0.004	34	-13.44	1.3e-13
baseline-acc.bth. (sh)	-0.016	0.005	34	-20.10	8.8e-19
baseline-acc.inv. (pe)	-0.004	0.002	34	-10.74	5.3e-11
baseline-acc.spc. (pe)	-0.006	0.004	34	-9.82	4.9e-10
baseline-acc.bth. (pe)	-0.010	0.004	34	-16.20	5.9e-16
baseline-acc.bth. (sh+pe)	-0.025	0.005	34	-26.22	1.9e-22
baseline-sem.inv. (sh)	-0.006	0.003	34	-11.09	2.3e-11
baseline-sem.spc. (sh)	-0.007	0.006	34	-7.60	1.3e-07
baseline-sem.bth. (sh)	-0.013	0.006	34	-13.60	9.6e-14
baseline-sem.inv. (pe)	-0.009	0.006	34	-8.41	1.5e-08
baseline-sem.spc. (pe)	-0.002	0.002	34	-4.50	6.9e-04
baseline-sem.bth. (pe)	-0.011	0.007	34	-9.31	1.7e-09
baseline-sem.bth. (sh+pe)	-0.022	0.008	34	-15.29	3.3e-15
baseline-acc.sem. (sh)	-0.027	0.005	34	-29.14	6.2e-24
baseline-acc.sem. (pe)	-0.020	0.007	34	-16.54	3.3e-16
baseline-acc.sem. (sh+pe)	-0.042	0.007	34	-32.46	1.8e-25
acc.inv. (sh)-acc.spc. (sh)	-0.002	0.006	34	-1.77	4.2e-01
acc.inv. (sh)-acc.bth. (sh)	-0.009	0.004	34	-12.61	7.7e-13
acc.spc. (sh)-acc.bth. (sh)	-0.007	0.004	34	-10.55	8.1e-11
acc.inv. (pe)-acc.spc. (pe)	-0.002	0.005	34	-2.00	3.2e-01
acc.inv. (pe)-acc.bth. (pe)	-0.006	0.003	34	-9.33	1.7e-09
acc.spc. (pe)-acc.bth. (pe)	-0.004	0.002	34	-10.15	2.1e-10
acc.inv. (sh)-acc.inv. (pe)	0.003	0.004	34	4.64	5.5e-04
acc.spc. (sh)-acc.spc. (pe)	0.004	0.005	34	4.59	5.9e-04
acc.bth. (sh)-acc.bth. (pe)	0.006	0.005	34	7.47	1.7e-07
acc.bth. (sh)-acc.bth. (sh+pe)	-0.008	0.003	34	-16.65	2.8e-16
acc.bth. (pe)-acc.bth. (sh+pe)	-0.015	0.004	34	-20.00	1.0e-18
sem.inv. (sh)-sem.spc. (sh)	-0.001	0.006	34	-0.88	3.9e-01
sem.inv. (sh)-sem.bth. (sh)	-0.007	0.005	34	-8.74	6.5e-09
sem.spc. (sh)-sem.bth. (sh)	-0.006	0.004	34	-8.99	3.4e-09
sem.inv. (pe)-sem.spc. (pe)	0.008	0.006	34	7.62	1.3e-07
sem.inv. (pe)-sem.bth. (pe)	-0.002	0.002	34	-6.28	5.2e-06
sem.spc. (pe)-sem.bth. (pe)	-0.010	0.007	34	-8.43	1.5e-08
sem.inv. (sh)-sem.inv. (pe)	-0.003	0.007	34	-2.55	1.2e-01
sem.spc. (sh)-sem.spc. (pe)	0.006	0.006	34	5.37	6.8e-05
sem.bth. (sh)-sem.bth. (pe)	0.002	0.008	34	1.48	5.9e-01
sem.bth. (sh)-sem.bth. (sh+pe)	-0.009	0.006	34	-9.20	2.2e-09
sem.bth. (pe)-sem.bth. (sh+pe)	-0.011	0.005	34	-12.52	9.1e-13

contrast	M	Std. Dev	df	t-value	p-value
acc.bth. (sh)-sem.bth. (sh)	0.003	0.008	34	2.08	3.2e-01
acc.bth. (pe)-sem.bth. (pe)	-0.001	0.008	34	-1.03	6.2e-01
acc.bth. (sh+pe)-sem.bth. (sh+pe)	0.003	0.011	34	1.40	5.1e-01
acc.bth. (sh)-acc.sem. (sh)	-0.011	0.005	34	-12.39	1.2e-12
sem.bth. (sh)-acc.sem. (sh)	-0.014	0.005	34	-17.50	6.3e-17
acc.bth. (pe)-acc.sem. (pe)	-0.010	0.006	34	-9.06	3.0e-09
sem.bth. (pe)-acc.sem. (pe)	-0.008	0.003	34	-15.04	5.3e-15
acc.sem. (sh)-acc.sem. (pe)	0.008	0.008	34	5.59	3.8e-05
acc.sem. (sh)-acc.sem. (sh+pe)	-0.014	0.005	34	-16.43	4.0e-16
acc.sem. (pe)-acc.sem. (sh+pe)	-0.022	0.005	34	-26.12	2.1e-22
acc.bth. (sh+pe)-acc.sem. (sh+pe)	-0.017	0.007	34	-13.38	1.5e-13
sem.bth. (sh+pe)-acc.sem. (sh+pe)	-0.020	0.005	34	-22.69	1.9e-20

S8 Table. Length of acoustic predictions does not explain RSA results. Results from between-item RSA regression models using $k = 1$ and restricting the temporal window to 0.0-0.48s to rule out prediction length as a confounding factor (see Conventional representational similarity analysis).
