# Supplementary material for: Sensory sharpening and semantic prediction errors unify competing models of predictive processing in human speech comprehension
Source: PLoS Biol. 2026 Jan 9;24(1):e3003588. doi: 10.1371/journal.pbio.3003588 (PMC12788694; doi:10.1371/journal.pbio.3003588)
Supplement: S9 Table — Results from contrasts in single-trial encoding models using pretrained transformers with d = 10. (PDF) [file pbio.3003588.s022.pdf]

contrast	M	Std. Dev.	df	t-value	p-value
inv. ac.-baseline	0.000076	0.000316	34	1.399752	1.000000
inv. sem.-baseline	0.000023	0.000350	34	0.376861	0.708619
inv. sem.-ac.	-0.000053	0.000508	34	-0.611232	1.000000
inv. bth.-baseline	0.000099	0.000438	34	1.311878	0.991752
inv. bth.-ac.	0.000023	0.000333	34	0.397016	1.000000
inv. bth.-sem.	0.000076	0.000352	34	1.258929	0.866525
spc. ac.-baseline	-0.000040	0.000329	34	-0.707456	0.968209
spc. sem.-baseline	0.000236	0.000230	34	5.982584	0.000005
spc. sem.-ac.	0.000276	0.000428	34	3.754684	0.002601
spc. bth.-baseline	0.000188	0.000431	34	2.545933	0.046801
spc. bth.-ac.	0.000228	0.000235	34	5.660051	0.000012
spc. bth.-sem.	-0.000048	0.000401	34	-0.696623	0.490774

S9 Table. Encoding models are robust to increased transformer dimensionality. Results from contrasts in single-trial encoding models using pretrained transformers with $d = 10$.
