# Supplementary material for: Sensory sharpening and semantic prediction errors unify competing models of predictive processing in human speech comprehension
Source: PLoS Biol. 2026 Jan 9;24(1):e3003588. doi: 10.1371/journal.pbio.3003588 (PMC12788694; doi:10.1371/journal.pbio.3003588)
Supplement: S10 Table — Results from contrasts in single-trial encoding models using control predictors derived from target words. (PDF) [file pbio.3003588.s023.pdf]

contrast	M	Std. Dev.	df	t-value	p-value
inv. ac.-baseline	0.000026	0.000799	34	0.191701	0.849117
inv. sem.-baseline	-0.000517	0.001237	34	-2.439485	0.080313
inv. sem.-ac.	-0.000544	0.001274	34	-2.488849	0.107237
inv. bth.-baseline	-0.000431	0.001368	34	-1.838238	0.224332
inv. bth.-ac.	-0.000458	0.001081	34	-2.469447	0.093559
inv. bth.-sem.	0.000086	0.000711	34	0.705434	0.970692
spc. ac.-baseline	-0.000175	0.000327	34	-3.116407	0.003709
spc. sem.-baseline	0.000961	0.000985	34	5.687480	0.000009
spc. sem.-ac.	0.001136	0.001106	34	5.989475	0.000005
spc. bth.-baseline	0.000760	0.000896	34	4.947531	0.000060
spc. bth.-ac.	0.000935	0.000943	34	5.778516	0.000008
spc. bth.-sem.	-0.000201	0.000314	34	-3.725773	0.001410

S10 Table. Results from encoding models are not contingent on transformers. Results from contrasts in single-trial encoding models using control predictors derived from target words.
