# Supplementary material for: Sensory sharpening and semantic prediction errors unify competing models of predictive processing in human speech comprehension
Source: PLoS Biol. 2026 Jan 9;24(1):e3003588. doi: 10.1371/journal.pbio.3003588 (PMC12788694; doi:10.1371/journal.pbio.3003588)
Supplement: S11 Table — Results from contrasts in single-trial encoding models in the follow-up task. (PDF) [file pbio.3003588.s024.pdf]

contrast	M	Std. Dev.	df	t-value	p-value
inc. inv. ac.-baseline	-0.000315	0.001721	34	-1.065444	5.883657e-01
inc. inv. sem.-baseline	0.001820	0.003506	34	3.026296	1.877703e-02
inc. inv. sem.-ac.	0.002134	0.003596	34	3.460802	8.825596e-03
inc. inv. bth.-baseline	0.001554	0.003933	34	2.304360	8.231825e-02
inc. inv. bth.-ac.	0.001869	0.003298	34	3.304056	1.125702e-02
inc. inv. bth.-sem.	-0.000265	0.001571	34	-0.984436	3.318550e-01
inc. spc. ac.-baseline	-0.001189	0.000724	34	-9.580910	2.070851e-10
inc. spc. sem.-baseline	-0.001074	0.002560	34	-2.446197	5.929342e-02
inc. spc. sem.-ac.	0.000116	0.002711	34	0.248509	8.052359e-01
inc. spc. bth.-baseline	-0.002037	0.002557	34	-4.646061	1.967241e-04
inc. spc. bth.-ac.	-0.000848	0.002522	34	-1.959992	1.164685e-01
inc. spc. bth.-sem.	-0.000963	0.000794	34	-7.071817	1.812218e-07
con. inv. ac.-baseline	-0.000387	0.001380	34	-1.634498	2.227622e-01
con. inv. sem.-baseline	0.001020	0.003020	34	1.969295	2.284476e-01
con. inv. sem.-ac.	0.001407	0.003301	34	2.484502	9.028907e-02
con. inv. bth.-baseline	0.000909	0.003185	34	1.663600	3.161534e-01
con. inv. bth.-ac.	0.001295	0.002896	34	2.608023	8.059220e-02
con. inv. bth.-sem.	-0.000111	0.001388	34	-0.467179	6.433524e-01
con. spc. ac.-baseline	-0.000813	0.000919	34	-5.157861	3.215765e-05
con. spc. sem.-baseline	0.002554	0.001775	34	8.392109	3.390330e-09
con. spc. sem.-ac.	0.003368	0.001905	34	10.309204	3.190655e-11
con. spc. bth.-baseline	0.001811	0.002108	34	5.007559	3.358951e-05
con. spc. bth.-ac.	0.002624	0.001774	34	8.622222	2.248759e-09
con. spc. bth.-sem.	-0.000744	0.000999	34	-4.342367	1.201464e-04

S11 Table. Double dissociation of semantic congruency and and prior specificity. Results from contrasts in single-trial encoding models in the follow-up task.
