# Supplementary material for: Sensory sharpening and semantic prediction errors unify competing models of predictive processing in human speech comprehension
Source: PLoS Biol. 2026 Jan 9;24(1):e3003588. doi: 10.1371/journal.pbio.3003588 (PMC12788694; doi:10.1371/journal.pbio.3003588)
Supplement: S12 Table — All stimulus pairs along with their corresponding context pairs used in the present study. (PDF) [file pbio.3003588.s025.pdf]

pair	contexts
Karte-Kette	tech-fashion
Anzüge-einzige	fashion-politics
Justiz-Notiz	politics-arts
Benutzer-Beschützer	tech-arts
Ballade-Panade	arts-food
Echtzeit-Bescheid	tech-politics
kürzen-würzen	fashion-food
Fährte-Konzerte	nature-arts
autokratisch-automatisch	politics-tech
Boot-Bit	nature-tech
Verwaltung-Vergeltung	politics-arts
kaputte-Kapuze	tech-fashion
Batterie-Fantasie	tech-arts
Brei-Schrei	food-arts
Agentur-Tastatur	politics-tech
segeln-Regeln	nature-politics
Olive-Motive	food-arts
Hitze-Witze	food-arts
Cloud-Braut	tech-fashion
Nacht-Recht	arts-politics
dörren-dürren	food-fashion
Brüste-Küste	fashion-nature
Regime-intim	politics-arts
Berg-Wert	nature-politics
Bühnen-grünen	arts-politics
schwimmen-Stimmen	nature-politics
Laser-Blazer	tech-fashion
schuften-duften	nature-food
Nadeln-radeln	fashion-nature
Wellen-Zellen	nature-tech
Platine-Gardine	tech-fashion
Tee-See	food-nature
betrogen-Beethoven	politics-arts
Münze-Künste	politics-arts
satt-Watt	food-tech
Professor-Prozessor	politics-tech
Stiefel-Staffel	fashion-arts
braune-Pflaume	fashion-food
Kopf-Topf	fashion-food
erkunden-erfunden	nature-arts
Zutaten-Flughafen	food-nature

pair	contexts
Lichter-Dichter	nature-arts
lecker-Hacker	food-tech
Daten-waten	tech-nature
Schal-Saal	fashion-arts
Feld-Geld	nature-politics
Schaum-Saum	food-fashion
Treibsand-Breitband	nature-tech
Meinungen-Leitungen	politics-tech
Mais-weiß	food-fashion
Kragen-fragen	fashion-politics
Hemd-Held	fashion-arts
suchen-Kuchen	nature-food
Spitzel-Pixel	politics-tech
Kopftuch-Kochbuch	fashion-food
Kayak-Cognac	nature-food
Wälder-Wähler	nature-politics
Volt-Wild	tech-nature
Schurke-Gurke	arts-food
Schmuck-Schluck	fashion-food

S12 Table. List of word- and context-pairs. All stimulus pairs along with their corresponding context pairs used in the present study.
