# Supplementary material for: Sensory sharpening and semantic prediction errors unify competing models of predictive processing in human speech comprehension
Source: PLoS Biol. 2026 Jan 9;24(1):e3003588. doi: 10.1371/journal.pbio.3003588 (PMC12788694; doi:10.1371/journal.pbio.3003588)
Supplement: Attachment — Submitted filename: HIB_response.pdf [file pbio.3003588.s026.pdf]

Sensory sharpening and semantic prediction errors unify competing models of predictive processing in communication: Peer Review

Fabian Schneider[1,2*] and Helen Blank[1,2]

[1]Institute for Systems Neuroscience, University Medical Centre Hamburg-Eppendorf, Martinistr. 52, Hamburg, 20246, Hamburg, Germany.
[2]Faculty of Psychology, Ruhr University Bochum, Universitätsstr. 150, Bochum, 44801, North-Rhine Westphalia, Germany.

*Corresponding author(s). E-mail(s): f.schneider@uke.de;
Contributing authors: helen.blank@rub.de;

1 General Response

We thank the four anonymous reviewers for their valuable constructive feedback. Based on their comments, we have added additional supplementary analyses and revised unclear sections and terminology in the manuscript. For your convenience, we mark original reviews in black, our responses in blue, and changes to the manuscript in green.

2 Response to reviewer #1

Ps learn to associate six faces with six contexts (food, fashion, technology, politics, arts, and nature) in an initial phase, where they are shown the face and told a one sentence summary of their interests. In the subsequent phase (EEG), they see a face, which is followed by an ambiguous word (two morphed). In part 2, the morph was replaced by a congruent or incongruent word. They were then shown feedback according to whether they had selected the congruent or incongruent option. Priors were fit to their responses from part 1 concerning the speaker-word associations. They fit the priors either as speaker-specific or speaker-general, i.e., across semantic contexts. Behaviour is biased towards priors. Neurally the picture is more complex. The signal is biased toward speaker-specific acoustic predictions at 165-1000 ms. And biased away from speaker-specific semantic predictions at 150-630 ms.

The manuscript is well written and the study appears interesting and well-designed. The behavioural effects seem a given in light of their design, but the neural effects are potentially very interesting. The analysis appears clever, although the details are difficult to follow - even as a relative specialist. Clarification is needed throughout. It also felt like different analysis strategies were used to test different hypotheses, and the logic here was hard to follow. Perhaps these were all specified in a prereg (in which case it would need less justification) but currently the reader will presumably need more justification for why each analysis for each question. If these issues could be fixed it would seem to make an interesting contribution to the literature - that neural signals are biased towards more sensory expectations and biased away from more semantic ones.

We thank the reviewer for their positive assessment of our manuscript and believe that our revisions clarify the issues they have raised and help justify the analyses presented here.

Specifics:

1.

That the design renders the behavioural effects a given. The authors test whether perception is pulled towards the context or pushed away from it. They find it is pulled towards it, and this effect increases over time. But the context-congruent option is highlighted in green as feedback. So the P's task is to select the word they heard, but given typical red-green colour associations, it seems they are told they are incorrect if they select the incongruent. So surely the opposite pattern of results was incredibly unlikely to materialise. Great if this point can be clarified and the conclusions altered accordingly.

We completely agree with the reviewer: We designed the task and feedback intentionally to make participants learn and apply the speaker-specific priors. Our aim was to investigate the neural mechanisms underlying speaker-specific

priors, and to ask whether/how/at which level speaker-specific priors alter speech processing. To save time during the experiment, we wanted to make the speaker priors as easy as possible. We have clarified this design choice and the resulting interpretation of the behavioural results in the manuscript:

Results 2.1, pp. 6, lines 114+

In experiment two (different $N_2 = 35$, see Participants), we directly replicated these behavioural findings (see Fig. 1c, Supplementary Table 2, Supplementary Fig. 1) while also recording EEG responses. Together, these behavioural results demonstrate that listeners can learn and apply speaker-specific semantic priors to aid disambiguation in speech perception when speaker-context associations are reinforced with speaker-specific feedback (see Fig. 1d). However, based on behavioural findings we cannot differentially test the computational mechanisms at play, as speaker-specific feedback rendered consistent behavioural errors unlikely and neural mechanisms underlying representation may diverge from simple behavioural read-outs[1].

Discussion, pp. 15, lines 388+

First, we demonstrate that listeners can apply speaker-specific semantic priors to disambiguate acoustically ambiguous words when speaker-context associations are consistently reinforced through feedback (see Fig. 1c). [...]

Discussion, pp. 16, lines 437+

Our task was carefully constructed to test facilitation of speech comprehension through speaker-specific semantic priors that were reinforced through explicit speaker-specific feedback. Therefore, it is also conceivable that other tasks demanding attention towards unexpected features, such as deviation detection, might shift low-level representations towards prediction error representations[2]. In complementary RSA analyses over decoded sensory representations, we also found some evidence of possible co-occurring prediction errors at the acoustic level that were, however, considerably weaker than sharpening responses (see Supplementary Figs. 7-8) and not apparent in the broadband EEG signal (see Figs. 3-4). This would be in line with the view that sharpening and prediction error computations co-occur in sensory regions at different laminae[3,4] where we might expect task- or context-based gain modulation[2] to dictate the dominance of sharpening or prediction error at the sensory level. We speculate that, even in this view, sharpening should dominate in natural, dialogic settings, in line with a synergistic account of sharpening and prediction errors[5].

2.

The authors could explain better why specific techniques/models were used for specific subsets/hypothesis. At first glance it feels like they have a separate encoding/decoding/or both (back2back)/ rsm approach and then look either at beta weights, correlation coefficients, or variance explained. This gets quite convoluted and it would help to have more support for those choices. If these choices were preregistered this would help, but if not, why these different strategies?

We thank the reviewer for pointing us to the missing explanations of techniques to address the respective hypotheses. We now clarify our two analysis pipelines.

> **Results, pp. 7, lines 148+**
>
> In a within-item cRSA approach, we computed cosine similarities of the reconstructed spectrograms from EEG signals following presentation of the identical morph in different contexts (e.g., *sea-tea|nature*, *sea-tea|food*, see Fig. 2b) with the real audio spectrograms of the corresponding original words (e.g., *sea*, *tea*) to obtain a similarity matrix of the composite sensory representations (i.e., sensory cRSM).
>
> We therefore measured within-item similarity, as opposed to between-item similarity as used in conventional RSA [6]. This approach was chosen because conventional RSA requires a commitment to specific mathematical formulations of the proposed computations for the construction of hypothesis RDMs [6]. However, theories of predictive processing allow for extremely high degrees of freedom in the precise formulation of their computations [7–11] which have been repeatedly criticised for hindering falsification [12–14]. Our experimental design, instead, aimed at differentiating between families of computations (see Fig. 1a,b,d,e): Do sensory representations drift towards (i.e., common to sharpening computations) or away from predictions (i.e., common to prediction error computations)? Anchoring decoded representational content to real constituent acoustic spectrograms allowed us to directly test for directional shifts in representational content given the same auditory stimulus in different speaker contexts (see Regressing composite representational similarity) rather than testing geometric signatures of specific mathematical formulations in second-order comparisons used in conventional RSA [6]. [...]
>
> **Results, pp. 10, lines 230+**
>
> [...] We examined whether the neural representations reflected sharpening or prediction error by using the best regression model's β-coefficients—i.e., the model incorporating both speaker-invariant

and -specific acoustic hypothesis cRSMs. Our hypothesis cRSMs were designed to reflect a pattern wherein more expected information is represented in positive values. In turn, due to the geometry of cosine similarities, this means that a negative sign inverts this pattern to represent more unexpected information (see Fig. 2e). Consequently, positive and negative coefficients in our models naturally correspond to sharpening and prediction error computations, respectively, which allowed us to directly test the directionality of the effect of acoustic predictions on sensory representations. [...]

Results, pp. 11, lines 268+

Building on our finding that speaker-specific semantic priors sharpen low-level sensory representations, we next examined whether prediction errors emerge at higher levels of the speech-processing hierarchy instead. To do this, we built encoding models (i.e., multivariate temporal response functions[15–17], mTRFs) of single-trial EEG signals that allowed us to probe signatures of acoustic and semantic prediction errors in the broadband EEG response. Critically, modeling broadband EEG responses rather than decoded sensory representations allowed us to probe whether prediction errors still co-occurred at, for example, the acoustic level as a separate information theoretic signal without shifting sensory representations. [...]

However, measuring prediction errors for morphs at levels higher than acoustics presents a unique challenge, as these stimuli are ill-defined at the phoneme and word level. To address this, we utilised recent advances in pretrained transformers[18], which allowed us to approximate general prediction error signals by feeding the raw audio of the morphs into these models and extracting 5-dimensional subspace projections of their layer activations[19,20]. Before feeding projected activations into encoding models, we verified that projected model activations were sensitive to general phonotactic, lexical and semantic prediction errors, and selected the best layer through back-to-back decoding[21]. This allowed us to disentangle the unique variance explained by each measure of prediction error in spite of existing correlations, in an independent auditory dataset (see Pretrained transformers as statistical surrogates, see also Supplementary Fig. 9).

[...]

To probe the spatiotemporal dynamics of speaker-specific semantic surprisal, we systematically eliminated the influence of speaker-specific semantic surprisal from the full model at temporal lags $\tau = \{-0.1, ..., 0.8\}s$, and measured the change in encoding performance of the full model over the reduced model relative to total encoding improvement of the full model over the baseline model, which yields

an estimate of the variance explained at any time point and channel (see Estimating spatiotemporal contributions of predictors). This knock-out procedure allowed us to directly test which temporal lags had a robust impact on encoding performance rather than having to rely on mTRF coefficients that may also reflect at least some degree of overfitting[22]. [...]

3.

As a specific, for the results in fig.2, what is the 'baseline' for each encoding improvement in 2D? Usually - and in the sense of a 'hierarchical' model - they would test baseline vs. 0, then acoustic vs. that baseline, and then semantic vs baseline + acoustic, which appears to be what they did according to model formulations in 2B (i.e. beta1 + beta2,3 + beta4,5). This however then rests on the assumption that the RDMs for acoustic and semantic invariant/specific are not highly correlated. If those RDMs look very similar, is this the most appropriate strategy? Clarification along these lines seems essential given the core claims about dissociations between acoustic and semantic predictions.

Many thanks for pointing this out. This hierarchical procedure is precisely what was employed here. We have clarified this procedure and that correlations between predictors were relatively low.

Results 2.2, pp. 7, lines 173+

To be able to assess the representational content in the sensory cRSM, we built two types of hypothesis cRSMs: [...] Correlations between hypothesis cRSMs were confirmed to be low, ruling out significant variance inflation (see Supplementary Fig. 5). We systematically regressed hypothesis cRSMs on sensory cRSMs in a hierarchical manner to assess the unique contributions of each hypothesis cRSM on sensory cRSMs, controlling for low-level acoustic features and general linguistic statistics that were independent of the semantic priors manipulated in our experiment (see Fig. 2b; see also Regressing composite representational similarity). [...]

Supplementary Materials 2.5, pp. 10

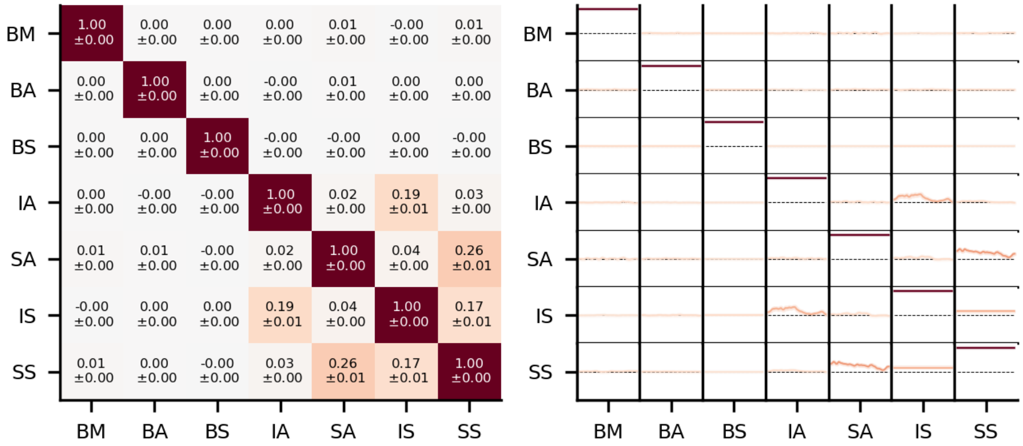

Supplementary Fig. 5 Correlations between all hypothesis cRSMs used in regressing sensory cRSMs for top-k predictions where $k = 5$. Hypothesis cRSMs included baseline morphs (BM), baseline acoustic predictions (BA), baseline semantic predictions (BS), speaker-invariant acoustic predictions (IA), speaker-specific acoustic predictions (SA), speaker-invariant semantic predictions (IS), and speaker-specific semantic predictions (SS). Baseline predictors were included in all models to control for acoustic properties of the morph (BM) as well as general acoustic (BA) and semantic predictions (BS) that were irrespective of our experiment. Left: Correlations were computed between all hypothesis cRSMs and averaged over time points. Individual cells contain means and standard errors computed over subjects. Right: Correlations were computed between all hypothesis cRSMs, but not averaged over time points. In each cell, time points (0.0-1.0s) are plotted on the x-axis, whereas correlations (-1 - 1) are plotted on the y-axis. Per cell, we plot chance level (dashed lines) and mean over subjects (solid lines) with confidence intervals indicated by shaded areas around means.

4.

It was unclear what a speaker-general prior would give us. The extent to which you associate anything with anything else? The theoretical significance of this formulation should be clarified.

Thanks for pointing out the lack of explanation. We have clarified the significance of speaker-invariant priors:

> **Results, pp. 3, lines 79+**
>
> To track participants' semantic expectations throughout the experiment, we computed trial-by-trial estimates of their semantic priors by fitting Gaussian distributions over semantic embeddings of the words they reported to hear through free-energy models in real-time (see Estimating real-time semantic models). To be able to dissociate learning of speaker-specific semantics and general semantics across the experiment (e.g., learning what kinds of semantic contexts are included in the experiment across all speakers) or, to a lesser degree, sequence effects (e.g., responses at trial t biassing responses at trial $t + 1$ irrespective of speaker-context), we fit two types of models: Firstly, modeling speaker-specific semantic priors, i.e. one prior per individual speaker, and, secondly, estimating speaker-invariant semantic priors, i.e., one general semantic prior across all six speaker contexts (see Fig. 1d).

5.

It's potentially very interesting if opposite effects are found according to acoustics vs semantics, but some further pointers would be helpful concerning how this resolves the conflicts in the literature. Are neural sharpening effects more common in sensory regions and prediction error effects in semantic processing locations? The authors consider how semantic error may be more useful for model updating but this presumably depends upon the level of the model. Interesting idea but it would seem to need further justification. In contrast the point about tasks (deviance detection) and the distinct neural effects they generate, was potentially very interesting. Maybe this latter point could receive more air time. Further justification for these claims given the current state of the literature would be very helpful.

Thank you for these thoughtful suggestions. We expanded on the interpretation of the observed opposing effects in our discussion and included more references to the current literature.

> **Discussion, pp. 15, lines 400+**

These findings align with recent refinements of Bayesian models of the brain, which propose that sharpening occurs at lower levels while prediction errors dominate higher levels of the neural hierarchy[5,23]. This nuanced view departs from traditional models of hPC, which posit that only prediction errors should be propagated within the hierarchy[10,24]. Instead, our results support a synergistic account, wherein sharpening enhances perceptual robustness while prediction errors may enable adaptive updating of internal models[5].

This interpretation is supported by prior work in speech perception demonstrating sharpening to occur at the sensory level[25,26]–though here explicit source modeling is lacking–and prediction errors in regions associated with higher-level processing such as phonology or semantics[27–31], though prediction errors were also identified in regions typically associated with acoustics exists[32]. Analogously, prior work in vision supports sharpening in sensory regions such as V1[33,34], but there are conflicting reports about prediction errors through-out the processing hierarchy[1,35,36]. More recent work in vision suggests that both sharpening and prediction errors may occur in deep and superficial laminae of V1, respectively[3,4], which may partly explain conflicting prior findings and complement the view that sharpening and prediction errors co-occur in cortical layers but dominate at different levels of the neural hierarchy[1,5]. However, laminar differences remain untested in speech comprehension, and cannot fully explain discrepancies between dominance of sharpening or prediction errors without assuming some kind of gain modulation thereof[2].

Here, we report empirical support for sharpening at the acoustic level (see Fig. 2e). Notably, the temporal extent of sharpening suggests that sharpening is not constrained to times of high uncertainty—for example, only for the disambiguating phonemes of a morph such as /t/ or /s/ for morph *tea-sea*—but occurs in parallel with the temporally unfolding acoustic signal instead. Sharpening at the acoustic level stands in contrast with previous findings emphasising the role of prediction error in speech processing[28,29,31]. This discrepancy may stem at least partly from methodological differences: whereas previous studies primarily analysed broadband EEG or BOLD responses, we employed speech decoding models[15] to isolate changes in low-level sensory representations. Supporting this view, evidence of sharpening in monologic contexts has previously been reported using similar sensory decoding approaches[25,26], albeit only for speech envelopes rather than low-level acoustic representations as captured with full spectrograms.

[...]

Yet, while these supplementary RSA results hint at weakly co-occurring acoustic prediction error at the sensory level (see Supplementary Figs. 7-8), we do not find evidence thereof in regressions of sensory cRSMs (see Fig. 2d) or encoding models of broadband EEG responses (see Fig. 3). In the case of cRSM regression, this is expected given that supplementary RSA results show acoustic sharpening to be considerably stronger than acoustic prediction errors (see Supplementary Fig. 7): When investigating the directionality of the representational shift overall, the stronger sharpening effect should dominate the weaker prediction error effect. That encoding models of broadband EEG responses do not support acoustic prediction errors (see Fig. 3d; Fig. 4) demonstrates the critical role that analysis granularity may play. As with sharpening, it is possible that multivariate sensory prediction errors are not easily detectable in commonly employed broadband encoding of univariate distance metrics[30,31]. In all, investigating predictive processing in natural speech by incorporating a wider array of methodologies–particularly, placing greater importance on representational content, laminar resolution of activity, and task-demands– may allow comparing and disentangling sharpening and prediction error computations at the acoustic level across monologic and dialogic contexts more thoroughly. Within regions, sharpening and prediction errors may be represented in deep and superficial layers, respectively[3,4], and across regions sharpening may dominate prediction errors at the sensory level[5] unless task-demands place greater importance on deviation detection[2].

Minor comments

1.

1. The manuscript contrasts 'predictive coding' with 'Bayesian' models. This is an unusual framing as predictive coding is often called a Bayesian brain theory. Predictive coding only emphasizes the processing of prediction errors in error units. In hypothesis units the opposite is true. I would therefore suggest replacing the name of the account specifying enhancement of an error signal as a predictive coding account. This could potentially lead to a misunderstanding of the theory, given the account only works because you have hypothesis and error units both playing key roles.

Thank you for highlighting this issue. We have amended the relevant sections to make this more clear.

Abstract

The human brain makes abundant predictions in speech comprehension that, in real-world conversations, depend on conversational

partners. Yet, tested models of predictive processing diverge on how such predictions are integrated with incoming speech: The brain may emphasise either expected information through sharpening or unexpected information through prediction error. [...]

Introduction, pp. 1, lines 17+

While prominent theories like hierarchical predictive coding (hPC) propose that higher-level priors, such as semantic expectations, generate lower-level predictions, such as as acoustic predictions, to facilitate perception and ambiguity resolution[10,24], how priors are applied at a mechanistic level is highly debated[7,23]: Empirically, prior work has reported enhancement of either expected components of the signal (sharpening), improving veridicality of representations[25,26,33,37], or unexpected parts of the signal (prediction error), improving informativeness of representations[28,29,31,38]. Yet, models of predictive processing typically assume distinct neuronal units for both sharpening and prediction error within regions[23,24]. Models tested in many empirical approaches, however, include only sharpening or prediction error components[25,26,31,37,38] and often do not track their dynamics jointly across time and levels of processing[28,29,33]. Therefore, it remains unclear how sharpening and prediction error computations are coordinated in function, over time, and across levels of the neural hierarchy[1,23]. Recent theoretical work predicts a hierarchical split: sharpening should dominate early sensory stages to stabilise perception, and prediction error should dominate later, higher-level stages to support adaptive updating of world models, but concrete evidence has been limited[5].

2.

It would be nice to have more information concerning how the alternative word was chosen. It is claimed that the phonetic differences are minimal, but not how these relate to other contexts. Was each target paired equally with a word from each of the other five contexts?

Indeed, the sampling here was balanced and designed in a highly controlled and symmetrical manner such that alternatives in one context served as targets in another context (e.g., *sea* in *sea-tea* as an alternative given *food* but as a target given *nature*). We have amended the methods to make this clearer:

Methods 4.2, pp. 20, lines 605+

[...] For each target word, one alternative word corresponding to one of the other five semantic contexts was chosen. Targets and alternatives were chosen such that phonemic differences would be minimal,

although not all formed minimal pairs (e.g., sea/tea in food/nature). We purposefully selected pairs of words with minimal acoustic differences, each of which fit into one of six semantic contexts. This allowed us to balance the stimuli symmetrically: Either member of a word pair was used as the target while the other served as the alternative, depending on the preceding context (e.g., target for *sea*-*tea*|*nature*: *sea*, but target for *sea*-*tea*|*food*: *tea*).

3 Response to reviewer #2

In "Sensory sharpening and semantic prediction errors unify competing models of predictive processing in communication," Schneider and Blank address a central debate in the predictive coding literature: is speech comprehension facilitated by the sharpening of expected content (as proposed in the Bayesian brain framework), or by the minimization of prediction errors through enhanced encoding of unexpected input (as posited by hierarchical predictive coding, hPC)? In a particularly elegant and methodologically rigorous study, the authors propose that these mechanisms are not mutually exclusive but can instead operate at different hierarchical levels of processing. Using a well-designed behavioral paradigm, they first demonstrate that listeners can learn and apply speaker-specific semantic priors. Participants learned associations between six distinct speaker faces and six unique semantic contexts. During the task, a speaker's face cued the semantic context, followed by an acoustically morphed word blending two words, only one of which was congruent with the speaker's semantic context. The results show that listeners relied on speaker-specific priors to disambiguate the perceived word.

To probe the neural underpinnings of this effect, the authors employed representational similarity analysis (RSA) and similarity encoding models. They reconstructed gammatone-filtered spectrograms from EEG recordings and found that correlations between reconstructed and actual audio spectrograms were significantly above chance. Next, they generated hypothesis RSMs based on the top-5 most probable acoustic predictions under each speaker-specific or -invariant prior and corresponding semantic predictions. They used encoding models to test which set of predictors best accounted for the similarity structure of the decoded EEG representations. The results revealed that both speaker-invariant and speaker-specific priors shaped EEG encoding of acoustic input, with a stronger contribution from speaker-specific priors. To differentiate sharpening from prediction error mechanisms, the authors analyzed the -coefficients of their best-fitting models. They showed positive coefficients for expected acoustic features under speaker-specific priors, indicating a sharpening effect: neural representations were biased toward predicted sensory input. They then asked whether prediction error signals emerge at higher levels of the processing hierarchy. To address this, they constructed single-trial encoding models of broadband EEG data, incorporating acoustic and semantic surprisal measures derived from large language models as control predictors. Only speaker-specific semantic surprisal significantly improved model performance, suggesting that prediction errors arise only at high-level semantic stages and not from general phonotactic or lexical violations. In a second EEG experiment, the authors tested the flexibility of semantic prior application by manipulating the congruency between prior expectations and the incoming speech signal. Participants heard vocoded but unmorphed words that were either highly congruent or incongruent with the speaker's semantic context. Behaviorally, incongruent trials resulted in longer reaction times,

indicating a processing cost. Encoding models revealed a double dissociation: in incongruent trials, only speaker-invariant surprisal improved encoding performance, whereas in congruent trials, only speaker-specific surprisal did.

Together, this study demonstrates that sharpening and prediction error mechanisms coexist in speech comprehension: low-level sensory sharpening occurs early and is guided by semantic priors, while prediction error signals emerge at later stages. I found the article very interesting, methodologically innovative, and well written. The study makes a contribution to the predictive coding literature by elegantly demonstrating how sharpening and prediction error mechanisms coexist in the processing hierarchies. I only have a few minor comments and suggestions for clarification:

We thank the reviewer for their positive assessment of our manuscript and have clarified relevant sections of the manuscript, as per the reviewer's thoughtful suggestions.

- Figure 1D: The correlation coefficients reported are relatively low. Some contextualization or discussion would be helpful to interpret the strength of these effects.

We agree with the reviewer and have added some context for the strength of these effects:

> **Results 2.2, pp. 8, lines 213+**
>
> [...] While differences in correlation coefficients reported here were relatively small, differences were highly consistent across participants (see Fig. 2d), and absolute differences were comparable to those commonly reported in RSA [1,6]. [...]

The authors chose to rely on raw EEG data. This limits insight into the specific neurophysiological features (e.g., time-frequency dynamics, phase-amplitude coupling, evoked responses) that carry sharpening or PE information. Including additional analyses based on TF decomposition, Hilbert envelopes or phase amplitude coupling might help clarify the underlying mechanisms of sharpening or PE. While I understand that addressing this would require substantial new analyses, the authors could consider discussing these potential features in the discussion section.

- Finally, the spatiotemporally resolved contributions of speaker-specific semantic surprisal (Figure 3C) and speaker-invariant semantic surprisal in incongruent trials (Figure 4D, top), as well as speaker-specific surprisal in congruent trials (Figure 4D, bottom), appear across all channels. This provides limited insight into the underlying neural networks involved.

Given these points, I believe the manuscript would benefit from a discussion or, if feasible, supplementary source-level and oscillatory analyses addressing the electrophysiological mechanisms and brain network dynamics

that may underlie these effects.

We agree that it would be highly interesting to have frequency-/coherence-resolved results at the source-level. However, there are considerable challenges here:

With respect to source-level analysis, we are fundamentally limited by EEG's spatial resolution. Source estimates derived from EEG data are extremely brittle even in the best case scenario, with expected localisation errors of several centimetres and similarly poor dispersion characteristics[39,40]. Here, we would face an additional problem because we did not collect structural MRIs of participants. Having to rely on template MRIs would likely render this an extremely optimistic lower bound. As such, we believe future studies will have to address this using MEG and, ideally, 3D-printed head masks.

With respect to TF decomposition and PAC, we do unfortunately also agree with the assessment of the reviewer that this would require substantial re-analyses that we believe to be outside the scope of this paper. For reference, the analyses presented in this paper take roughly two months of wall time when employing two NVIDIA RTX 5000 Ada GPUs. This presents a challenge because, for example, even splitting the analyses by low- and high-frequency oscillatory components only–e.g., $\leq 8Hz$ and $\geq 30Hz$ where sharpening and prediction errors should be apparent, respectively[41,42]–would add approximately four months of wall time.

Given these issues and the fact that our conclusions do not rely on assumptions about results of proposed analyses, we believe this will be more appropriate for a potential future re-analysis. As per the reviewer's suggestion, we now discuss and highlight this explicitly in the paper:

> **Discussion, pp. 17, lines 498+**
>
> With respect to both sharpening and prediction error computations reported here, two further avenues are of interest that fall beyond the scope of this paper: Firstly, source estimates would allow identification of their neural loci which we would expect to peak in A1, reflecting early auditory processing, and more distributed frontal and temporoparietal sources, reflecting later stages of semantic processing, respectively[43–45]. Secondly, theories of predictive coding have hypothesised distinct oscillatory signatures of predictions in lower and prediction errors in higher frequency bands[41,42] that may be investigated using MEG with individualised head casts to improve source estimates[46,47].

4 Response to reviewer #3

This manuscript presents research aimed at investigating the neural mechanisms underlying speech perception and how that perception is influenced by knowledge of an individual speaker's semantic priors. Specifically, the authors record EEG from participants as they listened to vocoded ambiguous (morphed) stimuli. Participants learned that certain speakers were more likely to speak about certain semantic topics and this influenced the perception of the ambiguous words. Using a combination of encoding/decoding modeling on the EEG and RSA the authors argue that the semantic priors sharpen the auditory representations of the ambiguous speech and lead to larger prediction errors at higher linguistic levels. They suggest that this leads to a more nuanced view of hierarchical predictive perception.

This was a well written manuscript that tackled an interesting question using a nice experimental design and sophisticated analyses. I thought the results were compelling and interesting and that the discussion was very reasonable. However, I do have a few comments - most of which center on the methods and the motivation for and description of those methods.

Many thanks for the positive assessment of our manuscript. As per the reviewer's helpful suggestions, we have now clarified the methodological choices, explanations and motivations in the manuscript.

Main comments:

1

I must admit to finding it somewhat difficult to understand the motivation for the multistep analysis. For example, for the question of whether acoustic representations are sharpened or whether they reflect prediction error, why not simply directly compare (via correlation) the reconstructed spectrograms for the morphs heard in the context of different speakers with the actual spectrograms of the stimuli making up those morphs? Would the reconstructed spectrogram being more highly correlated with tea than sea in one context and more highly correlated with sea than tea in the other (at least relatively) not allow you to quite directly compare the sharpening and prediction error accounts? Why the need to push it all through the RSA framework? I am not saying what you have done is incorrect. Just that it might be worth more clearly explaining the advantages of the approach you have chosen.

We thank the reviewer for highlighting the lack of explanation here. We respond to this comment and major comment two jointly. Please see the next response.

2

Following on from the previous comment, one of the main reasons why I wondered about the motivation for the RSA framework is that I also wondered about the specific implementation. In particular, the notion of building hypothesis RSMs based on a weighted average of the spectrograms of different words seemed very strange to me. Two words could have very similar semantic meaning and very different spectrograms. Averaging five (or more) spectrograms together seems certain to smear those spectrograms quite heavily and make the interpretation of the RSMs quite opaque, no? Moreover, I failed to appreciate why some words were more probable for a speaker than any other words. Was this just based on word frequency within category? And what about for speaker-independent, just the five most frequent words over all categories? Again, I think the motivation for the two-stage analysis could have been better. It is complicated and involves some assumptions and approaches that need to be justified and better explained in my opinion.

We follow the reviewer's suggestions and 1) explain why simply comparing morph sea-tea to the constituent words is inadequate, 2) motivate the choice of representational similarity in modeling different modalities, 3) explain how word probabilities are computed across (types of) priors, 4) provide more details, motivation and formal validation for top-k acoustic predictions, and 5) provide additional control analyses demonstrating the validity of top-k predictions:

Results, pp. 4, lines 91+

Critically, the same ambiguous morph (e.g., *sea-tea*) was presented in different speaker contexts (e.g., *nature-food*), allowing us to test whether perception and the underlying representation was pulled toward (i.e., sharpening) or away from the speaker priors (i.e., prediction error, see Fig. 1d,e). For example, an idealised sharpened representation of the morph between *sea*-tea given a speaker who was associated with the semantic context *nature* would be more similar to *sea* and related words, whereas the idealised prediction error representation would be more similar to *tea* and related words instead.

Results 2.2, pp. 7, lines 173+

To be able to assess the representational content in sensory cRSMs, we built two types of hypothesis cRSMs: Firstly, we built acoustic hypothesis cRSMs from spectrograms of clear words that were predictable from participants' speaker-specific and -invariant semantic priors. Specifically, we selected the five most probable words under a speaker prior and combined them into one probability-weighted audio spectrogram (for analysis of the influence of the number of predicted words, see Supplementary Figures 3-4, Supplementary Tables 4-5). This was done because predictions in the brain

are inherently distributional[24]: The brain cannot preminisce the target word at each trial, but predictions should reflect the distribution of predicted acoustic features instead (see Supplementary Methods 1.1). [...]

Supplementary Methods 1.1, pp. 1

Modeling acoustic expectations as top-*k* predictions

Our experimental design was tailored to address the question: How do expectations shape neural representations? Principally, a morph *sea-tea* given speaker context *nature* may be represented more like *sea* than *tea*–or vice versa. However, it is unlikely that the brain makes predictions that are this concrete, as it would require, for example, predicting *sea* at trial t ($sea\text{-}tea|nature$) but immediately thereafter *kayak* at trial $t+1$ ($kayak\text{-}cognac|nature$). In reality, the brain is assumed to make distributional predictions instead[24]. For example, the distributional expectation for *nature* $q(nature)$ may be:

$$q(nature) = \begin{bmatrix} p(sea|nature) \\ p(kayak|nature) \\ \vdots \\ p(tea|nature) \end{bmatrix} = \begin{bmatrix} 0.14 \\ 0.10 \\ \vdots \\ 0.02 \end{bmatrix} \tag{1}$$

where $p(word_i|context)$ is computed from semantic embeddings of individual words e_i and semantic priors obtained from free-energy models π:

$$p(w_i|\pi) = \frac{1}{2\bar{p}}\left(\frac{e_i \cdot \pi}{||e_i||\ ||\pi||} + 1\right) \qquad \text{where} \qquad \bar{p} = \sum_{i=0}^{k} p(w_i|\pi)$$

In other words, the probability of any word given a context is the normalised cosine similarity $\tilde{\angle} \in [0,1]$ between the semantic embedding of that word and the semantic embedding of the context obtained from free-energy models that evolve over the experiment, measuring their probability as a function of how similar a word is to a given prior. Note that, following prior work[48], we use cosine similarity rather than the likelihood of e_i under $\mathcal{N}(\mu_\pi, \sigma^2_\pi)$ as a computational simplification that works particularly well in high-dimensional semantic embeddings. Note also that context subsumes not only speaker-specific priors, but also speaker-invariant priors (i.e., context across the experiment).

Generally, we may then obtain an estimate of the prediction as:

$$\mathbb{E}_f(\pi) = \sum_{i=0}^{N} p(w_i|\pi) f(w_i) \qquad \text{where} \qquad \sum_{i=0}^{N} p(w_i|\pi) = 1$$

where f describes some feature we are interested in (for example, acoustic spectrograms), π refers to the speaker-specific or speaker-invariant semantic prior we are applying, and w_i is word i of N total words.

While this simplifies the prediction problem by assuming only a fixed vocabulary over stimuli included in the study, we can be reasonably certain that these truly reflect expected words. This is because, during stimulus creation, categories in semantic space were sampled densely, including those words that most closely aligned with any category. Therefore, these words should inherently reflect the most predictable words given any context.

When modeling semantic feature predictions, this approach could be approximated by comparing a morph sea-*tea* to its constituent words because semantic contexts reflect strong categorical structure whereby any word in *nature* is more similar to other words of *nature* than other contexts. Critically, words that are similar in meaning can be highly heterogeneous in acoustic features and vice versa (e.g., *dog*-*canine* or *bear*-*bare*). Consequently, modeling acoustic expectations requires a more explicit commitment to distributional predictions.

Therefore, here we model acoustic predictions as:

$$\mathbb{E}_s(\pi) = \sum_{i=0}^{k} p(w_k|\pi) s(w_k)$$

where s refers to spectrograms of individual words. While the resulting acoustic template $\mathbb{E}_s(\pi)$ may not correspond to a naturalistic waveform, the use of non-negative spectrogram magnitudes ensures that there is no destructive interference: Energy patterns of different words are mixed rather than cancelled, highlighting the spectral regularities between words as a function of their probability.

To illustrate the conservation of energy here, consider a decoder with a linear decision function:

$$f_c(x) = w_c^T x + b_c$$

with the expected top-k template:

$$\bar{x}_\pi = \mathbb{E}[x|\pi] = \sum_{w} p(w|\pi) x_w$$

Then for every feature c:

$$\begin{aligned} f_c(\bar{x}_\pi) &= w_c^T \left(\sum_w p(w|\pi) x_w \right) + b_c \\ &= \sum_w p(w|\pi) w_c^T x_w + b_c \\ &= \sum_w p(w|\pi)(w_c^T x_w + b_c) + \left(1 - \sum_w p(w|\pi)\right) b_c \\ &= \sum_w p(w|\pi) f_c(x_w) \qquad \text{since} \qquad \sum_w p(w|\pi) = 1 \end{aligned}$$

In other words, decoding features from top-k predictions (probability-weighted spectrogram templates) is formally equivalent to decoding features from each of the k individual words, weighted by their probabilities. This equality holds even with intercepts and affine transformations. An empirical validation thereof as well as an extension to representational similarity is provided in a Jupter notebook (topk_spectrogram_validation) available on GitHub[49].

Consequently, this approach allows us to create acoustic templates that reflect statistics of expected acoustic features that are predictable given a semantic prior. One caveat with this approach is the implicit simplifying assumption that words are predicted in full. Contrary to this assumption, we would expect the brain to make highly transient and auto-regressive predictions that are updated as the acoustic signal unfolds. For example, if we originally predict <crave>, <crane>, and <crowd>, once /kɹeɪ/ has been heard, <crowd> should be neglected–and predictions should be reweighted accordingly. This simplification was made for computational tractability.

Methods 4.9, pp. 26, lines 817+

Regressing composite representational similarity

To determine whether semantic expectations influenced the representation of morphed words at the acoustic level, we computed within-item composite representational similarity[6] matrices (cRSMs) comparing reconstructed spectrograms based on the recorded EEG data of the morphed stimuli with the real spectrograms of the audio files making up the respective morphs (e.g., /siː/ and /tiː/ for morph /_iː/). The observed cRSM for each morph n at time point t was defined as:

$$\mathrm{cRSM}(n,t) = \begin{bmatrix} f(/\_\mathrm{i\!:}/|\mathrm{nature}, /\mathrm{si\!:}/) & f(/\_\mathrm{i\!:}/|\mathrm{food}, /\mathrm{si\!:}/) \\ f(/\_\mathrm{i\!:}/|\mathrm{nature}, /\mathrm{ti\!:}/) & f(/\_\mathrm{i\!:}/|\mathrm{food}, /\mathrm{ti\!:}/) \end{bmatrix}$$

where f denotes cosine similarity computed over the frequency bins of gammatone spectrograms. This yielded a cRSM of size $120 \times 2 \times 2 \times 200$ (morph pairs $\times$ contexts $\times$ words $\times$ time points) per participant.

Note that this departs from conventional RSA [6] where RSMs are computed between items, which would have yielded an RSM of size $240 \times 240 \times 200$ (morphs $\times$ morphs $\times$ time points). Here, we opted for this approach because our research question and design pose a contrast at the within-item level: What is the direction in which predictions change the sensory representations of the same acoustic morph given different speaker contexts–towards or away from predictions (see Fig. 2)? Representational similarity allowed us to jointly model the influence of predictions in different modalities (i.e., acoustics and semantics), while anchoring decoded neural representations directly to real words allowed us to test this directly, without strong mathematical commitments to specific formulations underlying these computations that remain debated [7] but would have been required in a conventional second-order comparison through RSA [6]. Converging evidence from conventional RSA is presented in the supplement (see Supplementary Methods 1.3, Supplementary Fig. 7-8).

Results 2.2, pp. 10, lines 246+

We addressed several potential confounding factors: To rule out that our results depend on, firstly, the specific number of predicted words, we refit the best cRSM regression model while systematically varying the number of top-k predictions from 1 to 19, as there were 20 words per speaker. This analysis revealed significant stepwise improvements in out-of-sample performance, with diminishing returns for larger k (see Supplementary Fig. 3, Supplementary Table 4). We verified that our results were robust by repeating all previous analyses using $k = 19$ predictions (see Supplementary Fig. 4, Supplementary Table 5). Secondly, we confirmed that performance of top-k speaker-specific acoustic predictions depended on the specific top-k predictions at any trial and could not be explained equally well by top-k predictions that were not participant-specific (see Supplementary Methods 1.1-1.2, Supplementary Fig. 6, Supplementary Table 6). Further, we replicated our key finding of the dominance of acoustic sharpening at the sensory level in a conventional between-item RSA using $k = 1$ to rule out results being dependent on, thirdly, anchoring of hypothesis and sensory cRSMs to the same real spectrograms or, fourthly, averaging of spectrogram features (see Supplementary

Methods 1.3, Supplementary Fig. 7, Supplementary Table 7). Finally, we also verified that results do not depend on the length of acoustic predictions (see Supplementary Methods 1.3, Supplementary Fig. 8, Supplementary Table 8).

Supplementary Information 1.3, pp. 4

Conventional representational similarity analysis

To ensure that our finding of dominant sharpening at the acoustic level did not depend on the choice of analysis method (within-item composite RSM regression) or spectrogram averaging, we also conducted a conventional between-item representational similarity analysis (RSA)[6]. In this analysis we computed full representational similarity matrices (RSMs) for decoded sensory representations and all hypotheses from the original analysis (see Regressing composite representational similarity). Between-item RSA is typically used to compare representational geometries across systems[6]. As discussed in the manuscript (see Results 2.2), this requires a commitment to specific mathematical formulations of the computations of interest, which is challenging for predictive processing where a particularly high number of potential formulations exist[7–11]. To reduce degrees of freedom[12–14], we adopted two common mathematical formulations of sharpening and prediction error that are mathematically simplified by assuming no precision weighting[7,29]:

$$\text{sharpening(observation} \mid \text{expectation)} = \text{observation} \times \text{expectation}$$
$$\text{prediction error(observation} \mid \text{expectation)} = \text{observation} - \text{expectation}$$

Sensory RSMs were computed from cosine similarity between reconstructed spectrograms, and hypothesis RSMs were computed from: 1) morph spectrograms, 2) speaker-specific and speaker-invariant top-k acoustic predictions, and 3) speaker-specific and speaker-invariant semantic predictions. For this analysis we set $k = 1$ to avoid averaging across spectrograms. For semantic RSM computations, sharpening and prediction error formulations required disambiguation of morphs towards the target word (e.g., observation(sea-tea|nature) = sea) that were paired with their respective semantic priors (e.g., expectation(sea-tea|nature) = π(nature)); see Estimating real-time semantic models). In all, this yielded RSMs of size $240 \times 240 \times 200$ (morphs $\times$ morphs $\times$ time points) of which the upper triangles were used for regressions ($28,680 \times 200$).

To assess the influence of individual hypothesis RSMs, we used ridge regressions mapping from hypothesised RSMs X to observed

sensory RSMs y for each participant:

$$\hat{y}(t) = \beta_t X_t + \epsilon$$

where β_t are coefficients at time point t, X_t are hypothesis RSMs and $\hat{y}$ are predicted sensory RSMs. We solved for models β using PyTorch[50] and MVPy[51]:

$$\arg\min_{\beta_t} \sum_i (y_i - \beta_t^T X_t)^2 - \alpha_\beta ||\beta_t||^2$$

Ridge penalties α_β were optimised using leave-one-out cross-validation, testing 20 logarithmically spaced values between $1e^{-5} - 1e^{10}$[52]. All models were estimated with 5-fold cross-validation and 50 repetitions. Predictors were scaled to have zero mean and unit variance, based on training sets to prevent data leakage. Model performance was measured as out-of-sample Pearson correlation between $y(t)$ and $\hat{y}(t)$ in the held-out test sets, mirroring the procedure followed in our main analysis (see Regressing composite representational similarity).

Out-of-sample prediction performance between models was compared using two-sided paired t-tests with Bonferroni-Holm corrections, mirroring the original model comparison procedure in cRSM regressions (see Regressing composite representational similarity). For results from this analysis, see Supplementary Fig. 7 and Supplementary Table 7. For results from this analysis while restricting the temporal window to that of the shortest possible prediction $(0.0 - 0.48s)$, see Supplementary Fig. 8 and Supplementary Table 8.

Supplementary Information 2.7, pp. 12

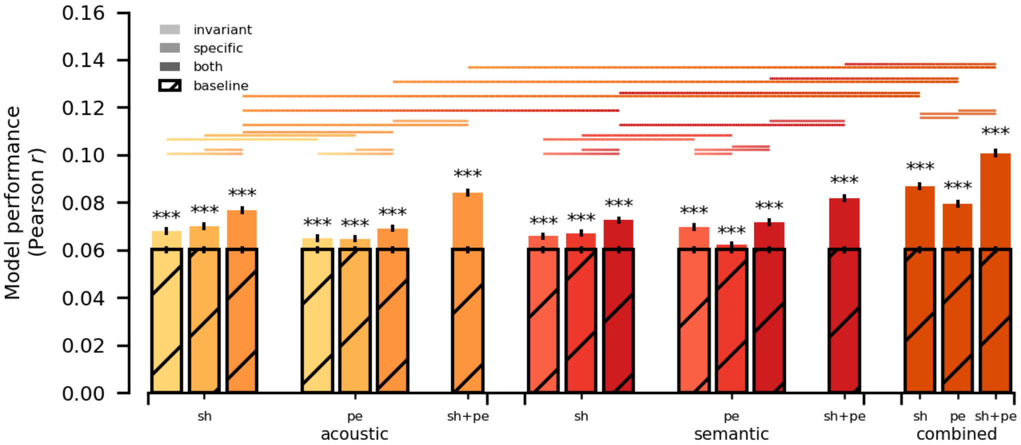

Supplementary Fig. 7 Between-item RSA regression models using $k = 1$ predictions to validate results using a conventional approach and without spectrogram averaging (see Conventional representational similarity analysis). Results confirm the dominance of acoustic sharpening (sh) over acoustic prediction errors (pe), as well as the dominance of acoustic over semantic sharpening. Unlike the within-item cRSM regression, this approach also shows modest additive variance explained by both acoustic prediction errors and semantic sharpening and prediction errors, though variance uniquely attributable to either is comparatively smaller (see Supplementary Table 6). Therefore, this does not change our interpretation that acoustic sharpening is the dominant computation at the sensory level, but suggests that multiple computations may co-occur at the same level to varying degrees. Individual bars represent group means, with black lines indicating 95%-confidence intervals around the mean. ***, **, * $p \leq 1e^{-3}$, $p \leq 1e^{-2}$, and $p \leq 5e^{-2}$ for model comparisons against baseline, respectively. Big bars between models indicate $p \leq 5e^{-2}$.

Supplementary Methods 1.2, pp. 3

Validating top-k acoustic predictions

If speaker-specific acoustic top-k predictions truly captured meaningful acoustic expectations of participants, disrupting these predictions should similarly deteriorate model performance.

To test this, we refit speaker-invariant and speaker-specific semantic priors (see Estimating real-time semantic models) for each participant while randomising their responses. This meant that semantic priors were now fit over the semantically coherent words in only 50% of trials. The remaining 50% of trials fell into one of five distinct alternative contexts (each with 10% chance). Because these were five disparate contexts that, jointly, were not semantically coherent, the newly estimated semantic priors still converged to coherent speaker-specific semantic priors that reflected a plausible estimate of the target context. Critically, however, they no longer represented the true semantic prior of any participant at any point in time.

Because this procedure preserved the overall semantic structure of priors but disrupted the degree to which participant-specific expectations were captured, we expected that model performance should be degraded for speaker-specific acoustic predictions, but that speaker-invariant acoustic predictions should remain largely unaffected given that experiment-specific statistics remain comparable.

To test this, we generated speaker-invariant and -specific acoustic and semantic top-k predictions from these foil priors. In accordance with our main analysis, we set $k = 5$. We then regressed foil cRSMs on sensory cRSMs, mirroring the procedure from our main findings (see Regressing composite representational similarity). We performed paired samples t-tests between performance of models using true and foil semantic priors with Bonferroni-Holm corrections applied.

Supplementary Information 2.6, pp. 11

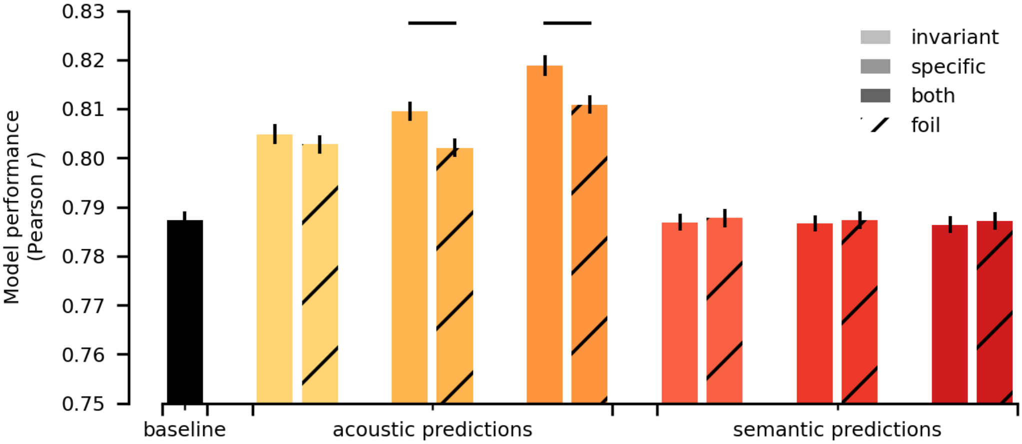

Supplementary Fig. 6 Validation analysis using foil priors that disrupted participant-specificity of semantic priors. To confirm that performance of top-k acoustic predictions depended on the trial-specific top-k predictions, we compared performance of predictions from the true speaker-invariant and -specific semantic priors with coherent but not trial-specific foil semantic priors in cRSM regressions (see Validating top-k acoustic predictions). In accordance with our main analysis, we set $k = 5$. As expected, this confirmed that speaker-specific acoustic predictions depended on the trial-specific predictions, indicating that they captured meaningful acoustic predictions. Note that, for visual clarity, we restricted the y-axis to a smaller range. Individual bars represent group means, with black lines indicating 95%-confidence intervals around the mean. Big bars between groups indicate $p \leq 5e^{-2}$.

Supplementary Information 2.8, pp. 13

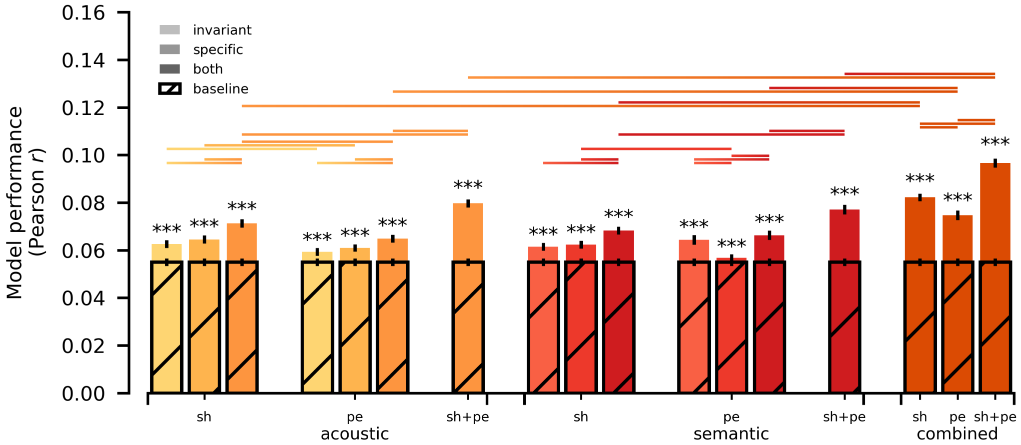

Supplementary Fig. 8 Between-item RSA regression models, setting $k = 1$ to avoid averaging and restricting the temporal window of the analysis to the shortest possible prediction (0.0-0.48s) such that differences in length could not affect results (see Conventional representational similarity analysis). Results corroborate the pattern observed without temporal restrictions (see Supplementary Fig. 5), ruling out prediction length as a confounding factor (see Supplementary Table 7). Individual bars represent group means, with black lines indicating 95%-confidence intervals around the mean. ***, **, * indicate $p \leq 1e^{-3}$, $p \leq 1e^{-2}$, and $p \leq 5e^{-2}$, respectively. Big bars between groups indicate $p \leq 5e^{-2}$.

3

Another question regarding the acoustic analysis: I struggled to understand from the methods section what was done for the speaker invariant analysis. I didn't see that explained in methods section 4.9 at all.

We thank the reviewer for highlighting the lack of explanation. We have made the following changes to the manuscript:

> **Methods 4.9, pp. 26, lines 843+**
>
> We formulated three core hypotheses to test how acoustic representations changed as a function of the speaker context (see Fig. 2b). For each prior-dependent hypothesis, predictions were derived from both speaker-specific and speaker-invariant priors. Both speaker-specific and -invariant hypotheses were therefore computed following the same procedure, with the only difference being the exact semantic prior distribution π–derived from speaker-specific or speaker-invariant free-energy models (see Estimating real-time semantic models)–that was used.
>
> [...]

Prior-dependent acoustic representation. If semantic priors influence the processing of the morphed words already at an acoustic stage, within-item similarity should reflect expected acoustics derived from the semantic priors. We estimated these expectations by computing the top-k predicted words:

$$\arg\max_{\text{word}} \sum_{i}^{k} p(\text{word}_i|\pi)$$

where π is a speaker-specific or -invariant semantic prior distribution derived from free-energy models (see Estimating real-time semantic models). Normalised probabilities were then used to compute a prior-weighted template from the top-k words that reflected expected acoustic features:

$$s(\text{predicted}|\pi) = \sum_{i}^{k} p(\text{word}_i|\pi)s(\text{word}_i)$$

[...]

Prior-dependent semantic representation. If semantic priors influence the processing of the morphed words at higher-level processing stages—for example, shifting sensory representations uniformly per semantic context—composite representational similarity should reflect direct comparisons between the prior distribution of the semantics given the speaker $\pi(\text{speaker})$ or speaker-invariant prior π (see Estimating real-time semantic models) and the word embeddings of the words making up the morph (e.g., *sea* and *tea*). [...]

4

The method section on the linguistic surprisal analysis (4.10) was confusing too I am afraid to say. It starts off by arguing that one can't really just calculate phonotactic/lexical/semantic surprisal for morphed stimuli - hence the need to use activations from different layers of wav2vec. But then it describes how you computed phonotactic/lexical/semantic surprisal. Why? Based on both words in the morph? Was the idea to see how the activations from different wav2vec layers relate to these different kinds of surprisal? And then to use the activations - or the feature-based predictions of the activations (or vice versa) - in the EEG analysis? It's all a bit opaque. And then in figures 3 and 4 we are only told about semantic surprisal - not sure where phonotactic and lexical surprisal went. Is the idea that you wanted to identify how the wav2vec activations related to semantic surprisal while removing the influence of phonotactic and lexical surprisal? We are also told the best layer is chosen. The best layer with respect to what feature? Semantic surprisal? It's very unclear what was done.

Thank you for drawing attention to this unclarity. We now describe more explicitly that this back-to-back decoding was performed over an independent narrative dataset where surprisal was well-defined which allowed us to select the layer that contained maximally represented surprisal measures jointly, allowing us to later use projected model activations to morphs as a control for general linguistic surprisal measures that were irrespective of our experiment and, consequently, not predictors of interest.

Methods 4.10, pp. 28, lines 912+

Pretrained transformers as statistical surrogates

For modeling of single-trial EEG responses, we required estimates of speaker-prior independent surprisal that are known to modulate neural responses[31]. Since phonotactic, lexical, and semantic surprisal are undefined for morphed words, we assessed how well these properties were captured by activations of pretrained transformers in a separate analysis.

To achieve this, an excerpt of a larger narrative (Alice in Wonderland, produced by the same speaker)–wherein phonotactic, lexical, and semantic surprisal were well-defined–was fed into ‘Wav2Vec2.0-large-xlsr-53-german‘[18,53] using PyTorch[50] and Transformers[54]. Layer-specific activations were extracted for narrative data. For computational efficiency, we performed layer-wise principle component analysis over narrative data and projected activations onto a 5-dimensional subspace. For layer selection and to confirm that the resulting activations included the relevant information theoretic measures, we built back-to-back decoding models[21]. In a first step, we built models mapping from layer activations X to observed measures y through a filter G:

$$\arg\min_{G} \sum_{i} (y_i - G^T X_i)^2 + \alpha_G ||G||^2$$

where y_i is a feature at trial i, X_i is the corresponding layer-specific activation and α_G is a regularisation parameter. These models were fit over the first half of the narrative data. The remaining half was then used to find the unique variance explained by each feature by mapping from decoded features $\hat{y}$ to observed features y through a filter H:

$$\arg\min_{H} \sum_{i} (y_i - H^T \hat{y}_i)^2 + \alpha_H ||H||^2$$

In both cases, we solved for filters G and H using sklearn[52] and PyTorch[50]. Optimal ridge penalties α_G, α_H were found using

leave-one-out cross-validation to test 20 logarithmically spaced values between $1e^{-5} - 1e^{10}$ [52]. This approach was employed for all time points from $0ms$-$500ms$ following phoneme onset, averaging over time to obtain an estimate of the causal contribution of each feature. To improve robustness, this procedure was repeated for 50 permutations of the narrative data [55]. Finally, we computed the proportion of variance explained by each feature to facilitate interpretation [56]:

$$R^2 = \frac{H}{\max_L \sum_i^F H_i}$$

where L are all layers of the LLM and F refers to all features. To identify the layer that was maximally sensitive to joint surprisal measures, we then ranked all layers per feature and took the geometric mean across features. The best layer was then selected by taking the median across permutations. For full results, please see Supplementary Fig. 9. Features were operationalised as follows:

[...]

For all features, surprisal was defined as $-\log_2 p$. Results revealed that, across an array of d-dimensional projections of model activations, layer 19 (transformer layer 12) was consistently sensitive to prior-independent phonotactic, lexical and semantic surprisal (see Supplementary Fig. 9).

Finally, we fed all morphs into the model and extracted model activations at layer 19. Model activations were projected into 5- and 10-dimensional subspaces and stored for later use as surrogate predictors that jointly controlled for measures of surprisal that were otherwise undefined for morphs when modeling single-trial EEG responses.

Methods 4.11, pp. 31, lines 996+

[...] Baseline models also included predictors controlling for general phonotactic, lexical and semantic surprisal. Generally, control predictors were derived from pretrained transformer activations (see Pretrained transformers as statistical surrogates). However, to rule out the possibility of performance of pretrained transformers given morphs confounding results, we also conducted a second set of analyses where control predictors were computed as direct measures of phonotactic, lexical and semantic surprisal by disambiguating the morph to the target word. [...]

5

BTW - is the speaker-specific vs speaker-independent semantic analysis also based somehow on the 5 most probable words?

For semantic predictions, we have an explicitly distributional prior π from free-energy models. Therefore, no top-k procedure was employed here (see Methods 4.9, 4.10, 4.11).

Minor comments:

1

It might be worth very briefly describing your GLMM in section 2.1 of the main body. The results are very difficult to appreciate without knowing what the model was trying to ask.

We agree with the reviewer and have added a brief explanation:

> **Results 2.1, pp. 5, lines 101+**
>
> In experiment one, participants ($N_1 = 35$, see Participants) were cued with a speaker's face and subsequently listened to a degraded spoken word (EEG part two, see Fig. 1b). We modeled the probability of reporting to have heard any word as a function of trial number, the word's probability given the speaker and remaining acoustic biases of morphs. Participants reported to hear [...]

2

Line 143 mentions "congruent words". Not sure what congruent means for this analysis/experiment.

Thank you for catching this. We have rephrased this:

> **Results, pp. 7, lines 183+**
>
> Secondly, we generated semantic hypothesis cRSMs by comparing the semantic embeddings of the constituent words (e.g., *sea*, *tea* for morph *sea*-*tea*) with the current estimates of the speaker-specific and speaker-invariant semantic priors from free-energy models (see Fig. 2b, see also Estimating real-time semantic models)

3

What was the motivation for the stimulus creation step described in the paragraph beginning on line 508? Why the need to minimize the geometric means of Rt and Ra?

We have clarified the purpose of this procedure:

> **Methods 4.2, pp. 21, lines 641+**

Next, for each target and alternative, the best semantic fits among the six candidate contexts were determined to find the subset of word pairs that maximised the coherence of each semantic context.

4

20 words per context and a total of sixty morphs. So, each word is morphed with 3 others?

Thank you for pointing us to this issue. We have clarified the phrasing:

Methods 4.2, pp. 21, lines 651+

As a result of this procedure, we obtained twenty words per context ($6 \times 20 = 120$ words overall), each of which was morphed with one other word, yielding a total of sixty morphs with two different noise profiles that showed no systematic perceptual biases (duration: $M = 867ms \pm 144ms$; for an overview, see Supplementary Fig. 13; Supplementary Table 12).

5

On lines 560-563... it seems you want find two words that are congruent and incongruent with a particular context. But you talk about a context_a and a context_b. Why?

Thanks for catching this. We have now clarified this:

Methods, 4.3, pp. 22, lines 689+

[...] Each pair contained one word strongly congruent with a speaker's semantic context (i.e., context_a) and another word strongly incongruent with a different context (i.e., context_b). To maximize the difference in semantic fit, word pairs were selected based on the following criterion:

$$\arg \max_{\text{word, context}} p(\text{word}_1|\text{context}_a) - p(\text{word}_2|\text{context}_b)$$

This ensured that word_1 was highly congruent with context_a, whereas word_2 was particularly incongruent with context_b. The sampling procedure also ensured that each context contained ten congruent-incongruent word pairs. This modification made the stimuli clearly identifiable as either congruent or incongruent, allowing us to test how speaker-specific priors are applied when word probability is either very high or very low for a given speaker.

5 Response to reviewer #4

The authors investigate how predictive coding and Bayesian models of speech comprehension might be reconciled. Using EEG with ambiguous speech presented in different speaker contexts, they test whether speaker-specific semantic priors sharpen sensory representations while prediction errors appear at higher linguistic levels. They propose that these mechanisms operate at distinct hierarchical stages of speech perception.

This is an ambitious paper, and some results - particularly regarding sharpening and the representational disambiguation of stimuli - are very interesting. However, because of methodological issues, I am not entirely convinced by the conclusions.

Before the details, I must note that the paper is written in an unclear and at times frustrating way. Many methodological choices are not well motivated, the use of technical terms often diverges from their standard usage, and explanations are unnecessarily dense, and cross-referencing is minimal. I spent much more time reviewing this paper than I normally would. The authors could do more to make it reader-friendly.

We thank the reviewer for their assessment that our manuscript presents ambitious and interesting work. To address their concerns, we followed the reviewer's suggestions and 1) aligned technical terms with standard usage, 2) clarified methodological explanations, 3) increased cross-referencing, and 4) introduced several additional control analyses.

General comments:

1. RSA-like approach

The authors describe their analysis as RSM-based, citing Kriegeskorte's RSA work, but their use of RSMs differs substantially from standard practice. This introduces unnecessary complexity and potential statistical bias.

In typical RSA, RSMs are square matrices containing pairwise distances between items in a model space. Here, RSMs are rectangular matrices reflecting *within-item* distances between decoded, real, and hypothesized spectrograms. This design may lose valuable information about dissimilarities across items.

The terminology is also misleading. For example, the matrix containing cosine similarities between real and decoded spectrograms is called the "sensory RSM," implying a uniform sensory representation rather than a composite of two representational formats.

Finally, all acoustic RSMs include distances from the real spectrogram. As a result, regression models (RSA analyses) measuring similarities between such RSMs may inflate scores because both are anchored to the same variable.

We thank the reviewer for these important comments. We agree with the reviewer that our method departs from the between-item metrics of traditional

RSA in favour of within-item information. We address their concerns by 1) clearly differentiating between conventional RSA and within-item composite RSA, 2) providing stronger theoretical motivation for this analytical choice, and 3) including supplementary results from a conventional RSA approach that converge with and deepen our findings and interpretations. Because of the volume of changes made to address this, we group the specific issue(s) addressed by any changes in the manuscript as follows into categories: (1) Differentiation of conventional RSA and composite RSA, (2) Motivation of analytical choices, and (3) Validation in conventional RSA.

(1) Differentiation

Introduction, pp. 2, lines 39+

We show that participants apply speaker-specific semantic priors when resolving ambiguities, reporting to hear the word consistent with the speaker's semantic context. Using stimulus reconstruction models[15] and within-item composite representational similarity[6] regression of recorded electroencephalography (EEG) responses, we show that these priors manifest in early sharpening of neural representations, with low-level acoustic content shifted toward the expected acoustic signal.

Results 2.2, pp. 6, lines 130+

To determine whether speaker-specific semantic priors influence low-level sensory representations early into the neural processing hierarchy, we employed an approach combining stimulus reconstruction models and within-item composite representational similarity analysis (cRSA; see Regressing composite representational similarity).

(1, 2, 3) Differentiation, Motivation, Validation

Results 2.2, pp. 7, lines 148+

In a within-item cRSA approach, we computed cosine similarities of the reconstructed spectrograms from EEG signals following presentation of the identical morph in different contexts (e.g., *sea-tea*|*nature*, *sea-tea*|*food*, see Fig. 2b) with the real audio spectrograms of the corresponding original words (e.g., sea, tea) to obtain a similarity matrix of the composite sensory representations (i.e., sensory cRSM).

We therefore measured within-item similarity, as opposed to between-item similarity as used in conventional RSA[6]. This approach was chosen because conventional RSA requires a commitment to specific mathematical formulations of the proposed computations for the construction of hypothesis RDMs[6]. However, theories of predictive processing allow for extremely high degrees of freedom in the precise

formulation of their computations[7–11] which have been repeatedly criticised for hindering falsification[12–14]. Our experimental design, instead, aimed at differentiating between families of computations (see Fig. 1a,b,d,e): Do sensory representations drift towards (i.e., common to sharpening computations) or away from predictions (i.e., common to prediction error computations)? Anchoring decoded representational content to real constituent acoustic spectrograms allowed us to directly test for directional shifts in representational content given the same auditory stimulus in different speaker contexts (see Regressing composite representational similarity) rather than testing geometric signatures of specific mathematical formulations in second-order comparisons used in conventional RSA[6]. While anchoring decoded representational content in real acoustic spectrograms may inflate absolute scores in cRSA regressions, relative explained variance between predictors on which our conclusions rely remain unbiassed.

To be able to assess the representatioanl content in sensory cRSMs, we built two types of hypothesis cRSMs: Firstly, we built acoustic hypothesis cRSMs from spectrograms of clear words that were predictable from participants' speaker-specific and -invariant semantic priors. Specifically, we selected the five most probable words under a speaker prior and combined them into one probability-weighted audio spectrogram (for analysis of the influence of the number of predicted words, see Supplementary Figures 3-4, Supplementary Tables 4-5). This was done because predictions in the brain are inherently distributional[24]: The brain cannot preminisce the target word at each trial, but predictions should reflect the distribution of predicted acoustic features instead (see Supplementary Methods 1.1). Secondly, we generated semantic hypothesis cRSMs by comparing the semantic embeddings of the constituent words (e.g., *sea*, *tea* for morph *sea*-*tea*) with the current estimates of the speaker-specific and speaker-invariant semantic priors from free-energy models (see Fig. 2b, see also Estimating real-time semantic models). Correlations between hypothesis cRSMs were confirmed to be low, ruling out significant variance inflation (see Supplementary Fig. 5). We systematically regressed hypothesis cRSMs on sensory cRSMs in a hierarchical manner to assess the unique contributions of each hypothesis cRSM on sensory cRSMs, controlling for low-level acoustic features and general linguistic statistics that were independent of the semantic priors manipulated in our experiment (see Fig. 2b; see also Regressing composite representational similarity). We hypothesised that speaker-specific semantic priors generate predictions at the acoustic level, i.e., acoustic predictions, which either pull sensory representations towards the expected signal—i.e., make the representation more similar to the expected words (sharpening)— or push

them away from it—i.e., render the representation more similar to the unexpected words (prediction error, see Fig. 1d,e).

cRSM regression revealed an effect of both speaker-specific and speaker-invariant semantic priors on acoustic representations. The sensory cRSMs were predicted more accurately when accounting for speaker-invariant and speaker-specific acoustic predictions (two-sided paired samples t-test, invariant-baseline: $t(34) = 22.40, p = 1.47e^{-20}$, specific-baseline: $t(34) = 25.12, p = 4.04e^{-22}$). Yet, speaker-specific acoustic predictions explained sensory cRSMs better than speaker-invariant acoustic predictions, indicating an increased reliance on speaker-specific information (two-sided paired samples t-test, specific-invariant: $t(34) = 4.18, p = 2.11e^{-3}$). Crucially, combining both types of acoustic cRSMs revealed their effects to be additive, with significantly better out-of-sample predictions than using either alone (two-sided paired samples t-test, both-baseline: $t(34) = 31.67, p = 2.33e^{-27}$, both-invariant: $t(34) = 17.87, p = 1.33e^{-17}$, both-specific: $t(34) = 15.03, p = 2.35e^{-15}$; see Fig. 2d). While differences in correlation coefficients reported here were relatively small, differences were highly consistent across participants (see Fig. 2d), and absolute differences were comparable to those commonly reported in RSA [1,6]. These findings suggest that predictions based on both speaker-invariant and speaker-specific semantic priors clearly shape the acoustic representations of heard words, though with a greater importance of speaker-specific priors.

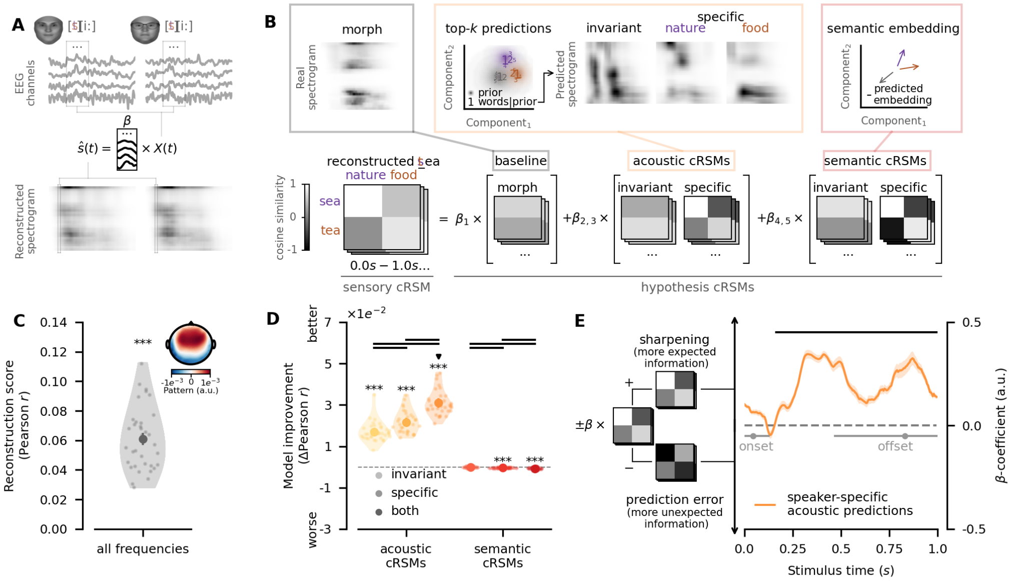

Fig. 2 **A** Stimulus reconstruction models were used to decode acoustic signals $\hat{s}$ from neural activity X during presentation of the same morph s given both of its potential speaker contexts (e.g., *nature* and *food* for morph *sea-tea*, see Stimulus reconstruction of morphs). This allowed us to compare sensory content of neural representations directly in a within-item composite representational similarity analysis (see Regressing composite representational similarity). **B** Composite sensory representational similarity matrices (cRSMs) were computed from the reconstructions of the identical morph given two speaker contexts (e.g., *nature* and *food*) and the two spectrograms of the audio files of the original word pair. A set of hypothesis cRSMs were computed from the raw morph, top-5 acoustic predictions, and the current semantic predictions. Hypothesis cRSMs were systematically regressed on sensory cRSMs to test which combination of predictors best explained the similarity structure of decoded neural representations (see Regressing composite representational similarity). **C** Stimulus reconstruction models performed significantly above chance level. The inlay shows the average pattern that reconstruction models decoded in z-scored EEG signals. **D** cRSM regression revealed that both speaker-invariant and speaker-specific acoustic cRSMs improved the similarity structure of decoded neural representations, and that purely semantic cRSMs failed to do so. Improvements in out-of-sample prediction are visualised relative to baseline models. Circles indicate group means with error bars on circles indicating 95%-confidence intervals thereof. Transparent dots indicate single subjects. ***, **, and * indicate $p \leq 1e^{-3}$, $p \leq 1e^{-2}$ and $p \leq 5e^{-2}$, respectively. Note that significantly worse performance indicates evidence against a specific model, not in favour of its antithesis. Bold black lines between groups indicate $p \leq 5e^{-2}$. All p-values were corrected using the Bonferroni-Holm procedure. **E** Time-resolved coefficients of speaker-specific acoustic cRSMs in regressions showed consistently positive signs, indicating that neural representations were sharpened towards the more expected information present in hypothesis cRSMs. Lines represent means, with shaded areas around lines indicating 95%-confidence intervals. Bold black lines indicate $p \leq 5e^{-2}$. Grey dots represent the median on- and offsets of the first and last phonemes, respectively, with 95%-highest density intervals around them.

To assess the specificity of these predictions to the acoustic level, we also tested whether purely semantic predictions would yield the same results. We entered the semantic embeddings used for top-5

predictions as semantic hypothesis cRSMs in the model, but omitted the step of generating specific acoustic predictions from these embeddings. Critically, purely semantic hypothesis cRSMs failed to improve out-of-sample predictions (two-sided paired samples t-test, invariant-baseline: $t(34) = -2.19, p = 0.25$, specific-baseline: $t(34) = -10.88, p = 1.66e^{-11}$, both-baseline: $M = t(34) = -7.93, p = 3.69e^{-8}$; see Fig. 2d). These results indicate that speaker-specific semantic priors alter sensory representations at the acoustic level.

Next, we asked how speaker-specific predictions are combined with the incoming sensory signals at the acoustic level. We examined whether the neural representations reflected sharpening or prediction error by using the best regression model's β-coefficients—i.e., the model incorporating both speaker-invariant and -specific acoustic hypothesis cRSMs. Our hypothesis cRSMs were designed to reflect a pattern wherein more expected information is represented in positive values. In turn, due to the geometry of cosine similarities, this means that a negative sign inverts this pattern to represent more unexpected information (see Fig. 2e). Consequently, positive and negative coefficients in our models naturally correspond to sharpening and prediction error computations, respectively, which allowed us to directly test the directionality of the effect of acoustic predictions on sensory representations. Cluster-based permutation tests revealed significant positive coefficients for speaker-specific acoustic predictions ($p \leq 8.00e^{-4}$) which corresponded to a cluster between $165ms$-$1000ms$ ($M = 0.22, s.d. = 0.04$; see Fig. 2e). These findings demonstrate that speaker-specific semantic priors sharpen neural representations by pulling neural representations towards the expected acoustic signal.

We addressed several potential confounding factors: To rule out that our results depend on, firstly, the specific number of predicted words, we refit the best cRSM regression model while systematically varying the number of top-k predictions from 1 to 19, as there were 20 words per speaker. This analysis revealed significant stepwise improvements in out-of-sample performance, with diminishing returns for larger k (see Supplementary Fig. 3, Supplementary Table 4). We verified that our results were robust by repeating all previous analyses using $k = 19$ predictions (see Supplementary Fig. 4, Supplementary Table 5). Secondly, we confirmed that performance of top-k speaker-specific acoustic predictions depended on the specific top-k predictions at any trial and could not be explained equally well by top-k predictions that were not participant-specific (see Supplementary Methods 1.2, Supplementary Fig. 6, Supplementary Table 6). Further, we replicated our key finding of the dominance of acoustic sharpening at the sensory level in a conventional between-item RSA

using $k = 1$ to rule out results being dependent on, thirdly, anchoring of hypothesis and sensory cRSMs to the same real spectrograms or, fourthly, averaging of spectrogram features (see Supplementary Methods 1.3, Supplementary Fig. 7, Supplementary Table 7). Finally, we also verified that results do not depend on the length of acoustic predictions (see Supplementary Methods 1.3, Supplementary Fig. 8, Supplementary Table 8).

(1, 2) Differentiation, Motivation

Methods 4.9, pp. 26, lines 817+

Regressing composite representational similarity

To determine whether semantic expectations influenced the representation of morphed words at the acoustic level, we computed within-item composite representational similarity[6] matrices (cRSMs) comparing reconstructed spectrograms based on the recorded EEG data of the morphed stimuli with the real spectrograms of the audio files making up the respective morphs (e.g., /siː/ and /tiː/ for morph /_iː/). The observed cRSM for each morph n at time point t was defined as:

$$\text{cRSM}(n,t) = \begin{bmatrix} f(/\_\text{iː}/|\text{nature}, /\text{siː}/) & f(/\_\text{iː}/|\text{food}, /\text{siː}/) \\ f(/\_\text{iː}/|\text{nature}, /\text{tiː}/) & f(/\_\text{iː}/|\text{food}, /\text{tiː}/) \end{bmatrix}$$

where f denotes cosine similarity computed over the frequency bins of gammatone spectrograms. This yielded a cRSM of size $120 \times 2 \times 2 \times 200$ (morph pairs $\times$ contexts $\times$ words $\times$ time points) per participant.

Note that this departs from conventional RSA[6] where RSMs are computed between items, which would have yielded an RSM of size $240 \times 240 \times 200$ (morphs $\times$ morphs $\times$ time points). Here, we opted for this approach because our research question and design pose a contrast at the within-item level: What is the direction in which predictions change the sensory representations of the same acoustic morph given different speaker contexts–towards or away from predictions (see Fig. 2)? Representational similarity allowed us to jointly model the influence of predictions in different modalities (i.e., acoustics and semantics), while anchoring decoded neural representations directly to real words allowed us to test this directly, without strong mathematical commitments to specific formulations underlying these computations that remain debated[7] but would have been required in a conventional second-order comparison through RSA[6]. Converging

evidence from conventional RSA is presented in the supplement (see Supplementary Methods 1.3, Supplementary Fig. 7-8).

We then generated hypothesis cRSMs to assess which representational structures best explained the observed cRSMs. We formulated three core hypotheses to test how acoustic representations changed as a function of the speaker context (see Fig. 2b). For each prior-dependent hypothesis, predictions were derived from both speaker-specific and speaker-invariant priors.

(1) Differentiation

Methods 4.9, pp. 27, lines 885+

Modeling approach. To test whether speaker context shifted participants' perception of the same acoustic morphs, we used ridge regressions mapping from hypothesis cRSMs X to sensory cRSMs y for each participant:

$$\hat{y}(t) = \beta_t X_t + \epsilon$$

where β_t are the coefficients at time point t. We constructed a temporally expanded design matrix D and temporally expanded outcomes Y, and solved for the model β using sklearn[52], sklearn-intelex[57] and PyTorch[50]:

$$\arg\min_{\beta} \sum_i (Y_i - \beta^T D_i)^2 + \alpha_\beta ||\beta||^2$$

Ridge penalties α_β were optimised using leave-one-out cross-validation to test 20 logarithmically spaced values between $1e^{-5} - 1e^{10}$[52]. Models were estimated using 5-fold cross-validation with 50 repetitions[55] and all outcomes and predictors were scaled to have zero mean and unit variance based on the training set to prevent data leakage. Out-of-sample performance was defined as the Pearson correlation between observed sensory cRSMs $y(t)$ and predicted sensory cRSMs $\hat{y}(t)$ in the held-out test set[58,59]. To ensure comparability across models, we normalised coefficients as:

$$\hat{\beta}_{j,t} = \frac{\beta_{j,t}}{\max_t \sum_j |\beta_j|}$$

Thus, $\hat{\beta}_j$ represents the relative contribution of predictor j to the variance explained[56], while meaningfully preserving signs.

Candidate models. Baseline models predicted sensory cRSMs from hypothesis cRSMs based on audio spectrograms of morphs as well as audio spectrograms and semantic embeddings from general language-specific expectations. We then performed step-wise inclusion of speaker-specific and speaker-invariant acoustic and semantic hypothesis cRSMs.

Statistical inference. Improvement in out-of-sample predictions for each hypothesis cRSM were tested using two-sided paired t-tests between candidate models, with Bonferroni-Holm corrections applied for multiple comparisons. Significance of temporally resolved variance explained by $\hat{\beta}$ was assessed through cluster-based permutation tests[60]. To compensate for the number of predictors, Bonferroni corrections were applied.

(3) Validation

Supplementary Information 1.3, pp. 4

Conventional representational similarity analysis

To ensure that our finding of dominant sharpening at the acoustic level did not depend on the choice of analysis method (within-item composite RSM regression) or spectrogram averaging, we also conducted a conventional between-item representational similarity analysis (RSA)[6]. In this analysis we computed full representational similarity matrices (RSMs) for decoded sensory representations and all hypotheses from the original analysis (see Regressing composite representational similarity). Between-item RSA is typically used to compare representational geometries across systems[6]. As discussed in the manuscript (see Results 2.2), this requires a commitment to specific mathematical formulations of the computations of interest, which is challenging for predictive processing where a particularly high number of potential formulations exist[7–11]. To reduce degrees of freedom[12–14], we adopted two common mathematical formulations of sharpening and prediction error that are mathematically simplified by assuming no precision weighting[7,29]:

$$\text{sharpening}(\text{observation} \mid \text{expectation}) = \text{observation} \times \text{expectation}$$
$$\text{prediction error}(\text{observation} \mid \text{expectation}) = \text{observation} - \text{expectation}$$

Sensory RSMs were computed from cosine similarity between reconstructed spectrograms, and hypothesis RSMs were computed from: 1) morph spectrograms, 2) speaker-specific and speaker-invariant top-k acoustic predictions, and 3) speaker-specific and speaker-invariant semantic predictions. For this analysis we set $k = 1$ to avoid averaging across spectrograms. For semantic RSM computations, sharpening and prediction error formulations required disambiguation of morphs towards the target word (e.g., observation(sea-tea|nature) = sea) that were paired with their respective semantic priors (e.g., expectation(sea-tea|nature) = π(nature)); see Estimating real-time semantic models). In all, this yielded RSMs

of size $240 \times 240 \times 200$ (morphs $\times$ morphs $\times$ time points) of which the upper triangles were used for regressions $(28,680 \times 200)$.

To assess the influence of individual hypothesis RSMs, we used ridge regressions mapping from hypothesised RSMs X to observed sensory RSMs y for each participant:

$$\hat{y}(t) = \beta_t X_t + \epsilon$$

where β_t are coefficients at time point t, X_t are hypothesis RSMs and $\hat{y}$ are predicted sensory RSMs. We solved for models β using PyTorch[50] and MVPy[51]:

$$\arg\min_{\beta_t} \sum_i (y_i - \beta_t^T X_t)^2 - \alpha_\beta ||\beta_t||^2$$

Ridge penalties α_β were optimised using leave-one-out cross-validation, testing 20 logarithmically spaced values between $1e^{-5} - 1e^{10}$[52]. All models were estimated with 5-fold cross-validation and 50 repetitions. Predictors were scaled to have zero mean and unit variance, based on training sets to prevent data leakage. Model performance was measured as out-of-sample Pearson correlation between $y(t)$ and $\hat{y}(t)$ in the held-out test sets, mirroring the procedure followed in our main analysis (see Regressing composite representational similarity).

Out-of-sample prediction performance between models was compared using two-sided paired t-tests with Bonferroni-Holm corrections, mirroring the original model comparison procedure in cRSM regressions (see Regressing composite representational similarity). For results from this analysis, see Supplementary Fig. 7 and Supplementary Table 7. For results from this analysis while restricting the temporal window to that of the shortest possible prediction $(0.0 - 0.48s)$, see Supplementary Fig. 8 and Supplementary Table 8.

Supplementary Information 2.7, pp. 12

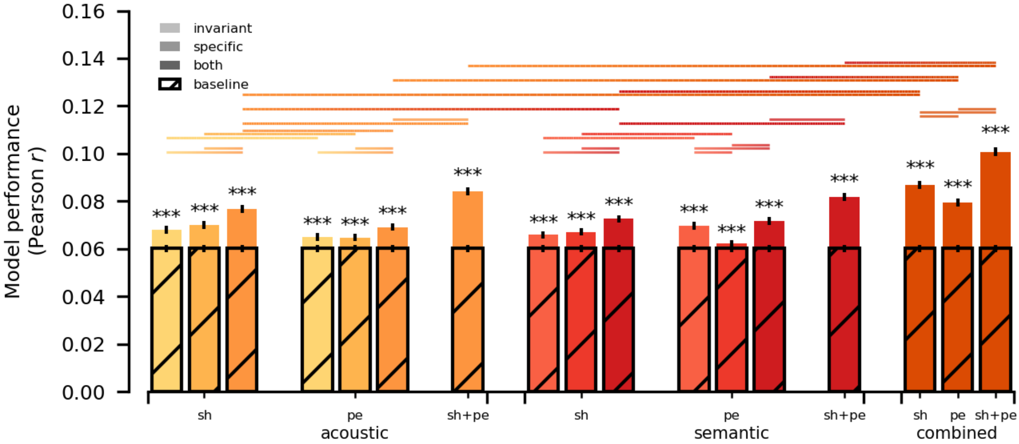

Supplementary Fig. 7 Between-item RSA regression models using $k = 1$ predictions to validate results using a conventional approach and without spectrogram averaging (see Conventional representational similarity analysis). Results confirm the dominance of acoustic sharpening (sh) over acoustic prediction errors (pe), as well as the dominance of acoustic over semantic sharpening. Unlike the within-item cRSM regression, this approach also shows modest additive variance explained by both acoustic prediction errors and semantic sharpening and prediction errors, though variance uniquely attributable to either is comparatively smaller (see Supplementary Table 6). Therefore, this does not change our interpretation that acoustic sharpening is the dominant computation at the sensory level, but suggests that multiple computations may co-occur at the same level to varying degrees. Individual bars represent group means, with black lines indicating 95%-confidence intervals around the mean. ***, **, * $p \leq 1e^{-3}$, $p \leq 1e^{-2}$, and $p \leq 5e^{-2}$ for model comparisons against baseline, respectively. Big bars between models indicate $p \leq 5e^{-2}$.

Discussion, pp. 16, lines 423+

Here, we report empirical support for sharpening at the acoustic level (see Fig. 2e). Notably, the temporal extent of sharpening suggests that sharpening is not constrained to times of high uncertainty—for example, only for the disambiguating phonemes of a morph such as /t/ or /s/ for morph *tea*-*sea*—but occurs in parallel with the temporally unfolding acoustic signal instead. Sharpening at the acoustic level stands in contrast with previous findings emphasising the role of prediction error in speech processing[28,29,31]. This discrepancy may stem at least partly from methodological differences: whereas previous studies primarily analysed broadband EEG or BOLD responses, we employed speech decoding models[15] to isolate changes in low-level sensory representations. Supporting this view, evidence of sharpening in monologic contexts has previously been reported using similar sensory decoding approaches[25,26], albeit only for speech envelopes rather than low-level acoustic representations as captured with full spectrograms.

Our task was carefully constructed to test facilitation of speech comprehension through speaker-specific semantic priors that were reinforced through explicit speaker-specific feedback. Therefore, it is also conceivable that other tasks demanding attention towards unexpected features, such as deviation detection, might shift low-level representations towards prediction error representations[2]. In complementary RSA analyses over decoded sensory representations, we also found some evidence of possible co-occurring prediction errors at the acoustic level that were, however, considerably weaker than sharpening responses (see Supplementary Figs. 7-8) and not apparent in the broadband EEG signal (see Figs. 3-4). This would be in line with the view that sharpening and prediction error computations co-occur in sensory regions at different laminae[3,4] where we might expect task- or context-based gain modulation[2] to dictate the dominance of sharpening or prediction error at the sensory level. We speculate that, even in this view, sharpening should dominate in natural, dialogic settings, in line with a synergistic account of sharpening and prediction errors[5].

2. Prior-weighted averages

I am not convinced by the idea of prior-weighted spectrograms. It seems implausible that averaging the spectrograms of the top 5 (or 19) exemplars of a semantic category would yield a meaningful acoustic signal, given that the mapping between sound and meaning is arbitrary. I'd expect that predicted acoustics will average out. Such averaging makes more sense for semantic embeddings, where words in the same category cluster in vector space.

Also, how are spectrograms of different word lengths averaged? Could length differences alone drive the results? A long spectrogram could correlate better simply because the averaged template includes a long word.

For these reasons, I find cosine-distance analyses to these averaged spectrograms questionable.

Thank you for raising this critical point. We now address this more explicitly in the manuscript and supplement by:

1) demonstrating formally that top-k spectrogram averaging produces meaningful spectral templates.

2) showing that, even in the case of $k = 1$ where no averaging occurs, results reported here persist.

3) confirming that length of predictions does not confound these results.

4) validating this top-k procedure empirically by showing that employing plausible semantic priors that were, crucially, not participant-specific to generate top-k acoustic predictions significantly degrades model performance, indicating that top-k acoustic predictions truly do capture participant-specific spectral expectations.

As the volume of changes is relatively substantial, we again categorise changes as follows: (1) Formal validation, and (2) Empirical validation. We have amended the manuscript as follows:

(1) Formal validation

Supplementary Methods 1.1, pp. 1

Modeling acoustic expectations as top-k predictions

Our experimental design was tailored to address the question: How do expectations shape neural representations? Principally, a morph *sea-tea* given speaker context *nature* may be represented more like *sea* than *tea*–or vice versa. However, it is unlikely that the brain makes predictions that are this concrete, as it would require, for example, predicting *sea* at trial t ($sea\text{-}tea|nature$) but immediately thereafter *kayak* at trial $t + 1$ ($kayak\text{-}cognac|nature$). In reality, the brain is assumed to make distributional predictions instead[24]. For example, the distributional expectation for *nature* $q(nature)$ may be:

$$q(nature) = \begin{bmatrix} p(sea|nature) \\ p(kayak|nature) \\ \vdots \\ p(tea|nature) \end{bmatrix} = \begin{bmatrix} 0.14 \\ 0.10 \\ \vdots \\ 0.02 \end{bmatrix} \tag{2}$$

where $p(word_i|context)$ is computed from semantic embeddings of individual words e_i and semantic priors obtained from free-energy

models π:

$$p(w_i|\pi) = \frac{1}{2\bar{p}}\left(\frac{e_i \cdot \pi}{||e_i||\ ||\pi||} + 1\right) \qquad \text{where} \qquad \bar{p} = \sum_{i=0}^{k} p(w_i|\pi)$$

In other words, the probability of any word given a context is the normalised cosine similarity $\tilde{\angle} \in [0, 1]$ between the semantic embedding of that word and the semantic embedding of the context obtained from free-energy models that evolve over the experiment, measuring their probability as a function of how similar a word is to a given prior. Note that, following prior work[48], we use cosine similarity rather than the likelihood of e_i under $\mathcal{N}(\mu_\pi, \sigma_\pi^2)$ as a computational simplification that works particularly well in high-dimensional semantic embeddings. Note also that context subsumes not only speaker-specific priors, but also speaker-invariant priors (i.e., context across the experiment).

Generally, we may then obtain an estimate of the prediction as:

$$\mathbb{E}_f(\pi) = \sum_{i=0}^{N} p(w_i|\pi) f(w_i) \qquad \text{where} \qquad \sum_{i=0}^{N} p(w_i|\pi) = 1$$

where f describes some feature we are interested in (for example, acoustic spectrograms), π refers to the speaker-specific or speaker-invariant semantic prior we are applying, and w_i is word i of N total words.

While this simplifies the prediction problem by assuming only a fixed vocabulary over stimuli included in the study, we can be reasonably certain that these truly reflect expected words. This is because, during stimulus creation, categories in semantic space were sampled densely, including those words that most closely aligned with any category. Therefore, these words should inherently reflect the most predictable words given any context.

When modeling semantic feature predictions, this approach could be approximated by comparing a morph sea-*tea* to its constituent words because semantic contexts reflect strong categorical structure whereby any word in *nature* is more similar to other words of *nature* than other contexts. Critically, words that are similar in meaning can be highly heterogeneous in acoustic features and vice versa (e.g., *dog*-*canine* or *bear*-*bare*). Consequently, modeling acoustic expectations requires a more explicit commitment to distributional predictions.

Therefore, here we model acoustic predictions as:

$$\mathbb{E}_s(\pi) = \sum_{i=0}^{k} p(w_k|\pi) s(w_k)$$

where s refers to spectrograms of individual words. While the resulting acoustic template $\mathbb{E}_s(\pi)$ may not correspond to a naturalistic waveform, the use of non-negative spectrogram magnitudes ensures that there is no destructive interference: Energy patterns of different words are mixed rather than cancelled, highlighting the spectral regularities between words as a function of their probability.

To illustrate the conservation of energy here, consider a decoder with a linear decision function:

$$f_c(x) = w_c^T x + b_c$$

with the expected top-k template:

$$\bar{x}_\pi = \mathbb{E}[x|\pi] = \sum_w p(w|\pi) x_w$$

Then for every feature c:

$$\begin{aligned} f_c(\bar{x}_\pi) &= w_c^T \left(\sum_w p(w|\pi) x_w \right) + b_c \\ &= \sum_w p(w|\pi) w_c^T x_w + b_c \\ &= \sum_w p(w|\pi)(w_c^T x_w + b_c) + \left(1 - \sum_w p(w|\pi) \right) b_c \\ &= \sum_w p(w|\pi) f_c(x_w) \qquad \text{since} \qquad \sum_w p(w|\pi) = 1 \end{aligned}$$

In other words, decoding features from top-k predictions (probability-weighted spectrogram templates) is formally equivalent to decoding features from each of the k individual words, weighted by their probabilities. This equality holds even with intercepts and affine transformations. An empirical validation thereof as well as an extension to representational similarity is provided in a Jupter notebook (topk_spectrogram_validation) available on GitHub[49].

Consequently, this approach allows us to create acoustic templates that reflect statistics of expected acoustic features that are predictable given a semantic prior. One caveat with this approach is the implicit simplifying assumption that words are predicted in full. Contrary to this assumption, we would expect the brain to make highly transient and auto-regressive predictions that are updated as the acoustic signal unfolds. For example, if we originally predict <crave>, <crane>, and <crowd>, once /kɹeɪ/ has been heard, <crowd>

should be neglected–and predictions should be reweighted accordingly. This simplification was made for computational tractability.

Methods 4.9, pp. 27, lines 857+

Prior-dependent acoustic representations. If semantic priors influence the processing of the morphed words already at an acoustic stage, within-item similarity should reflect expected acoustic features derived from the semantic priors. We estimated these expected features as acoustic templates by computing the top-k predicted words:

$$\arg\max_{\text{word}} \sum_{i}^{k} p(\text{word}_i|\pi)$$

where π is a speaker-specific or -invariant semantic prior distribution derived from free-energy models (see Estimating real-time semantic models). Normalised probabilities were then used to compute a prior-weighted template from top-k words that reflected expected acoustic features:

$$s(\text{predicted}|\pi) = \sum_{i}^{k} p(\text{word}_i|\pi)s(\text{word}_i)$$

Since spectrogram magnitudes are inherently non-negative, there is no destructive interference here. Instead, this weighted procedure aggregates spectral energy from individual word predictions that together form an expected template that reflects predicted acoustic features (see Supplementary Methods 1.1-1.2; see also Supplementary Fig. 6). Expected acoustic templates were then compared with the respective real spectrograms making up the morph (e.g., /siː/ and /tiː/).

(2) Empirical validation

Results 2.2, pp. 10, lines 246+

We addressed several potential confounding factors: [...] Secondly, we confirmed that top-k acoustic predictions captured meaningful acoustic expectations of participants that could not be explained equally well by top-k predictions that were not participant-specific (see Supplementary Methods, Supplementary Fig. 6, Supplementary Table 6). Further, we replicated our key finding of the dominance of acoustic sharpening at the sensory level in a conventional between-item RSA using $k = 1$ to rule out results being dependent on, thirdly, anchoring of hypothesis and sensory cRSMs to the same real spectrograms or, fourthly, averaging of spectrogram features (see Supplementary Methods 1.3, Supplementary Fig. 7, Supplementary Table 7). Finally, we also verified that results do not depend on the length

of acoustic predictions (see Supplementary Methods, Supplementary Fig. 8, Supplementary Table 8).

Supplementary Methods 1.2, pp. 3

Validating top-k acoustic predictions

If speaker-specific acoustic top-k predictions truly captured meaningful acoustic expectations of participants, disrupting these predictions should similarly deteriorate model performance.

To test this, we refit speaker-invariant and speaker-specific semantic priors (see Estimating real-time semantic models) for each participant while randomising their responses. This meant that semantic priors were now fit over the semantically coherent words in only 50% of trials. The remaining 50% of trials fell into one of five distinct alternative contexts (each with 10% chance). Because these were five disparate contexts that, jointly, were not semantically coherent, the newly estimated semantic priors still converged to coherent speaker-specific semantic priors that reflected a plausible estimate of the target context. Critically, however, they no longer represented the true semantic prior of any participant at any point in time.

Because this procedure preserved the overall semantic structure of priors but disrupted the degree to which participant-specific expectations were captured, we expected that model performance should be degraded for speaker-specific acoustic predictions, but that speaker-invariant acoustic predictions should remain largely unaffected given that experiment-specific statistics remain comparable.

To test this, we generated speaker-invariant and -specific acoustic and semantic top-k predictions from these foil priors. In accordance with our main analysis, we set $k = 5$. We then regressed foil cRSMs on sensory cRSMs, mirroring the procedure from our main findings (see Regressing composite representational similarity). We performed paired samples t-tests between performance of models using true and foil semantic priors with Bonferroni-Holm corrections applied.

Supplementary Information 2.6, pp. 11

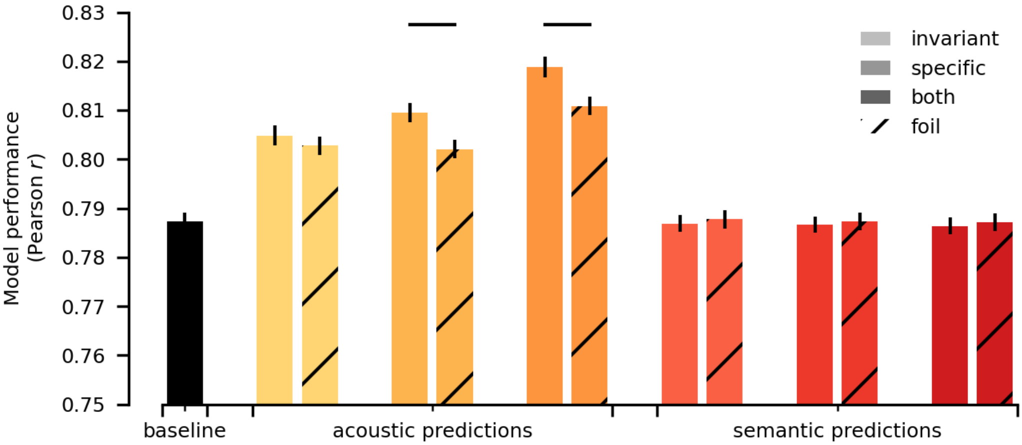

Supplementary Fig. 6 Validation analysis using foil priors that disrupted participant-specificity of semantic priors. To confirm that performance of top-k acoustic predictions depended on the trial-specific top-k predictions, we compared performance of predictions from the true speaker-invariant and -specific semantic priors with coherent but not trial-specific foil semantic priors in cRSM regressions (see Validating top-k acoustic predictions). In accordance with our main analysis, we set $k = 5$. As expected, this confirmed that speaker-specific acoustic predictions depended on the trial-specific predictions, indicating that they captured meaningful acoustic predictions. Note that, for visual clarity, we restricted the y-axis to a smaller range. Individual bars represent group means, with black lines indicating 95%-confidence intervals around the mean. Big bars between groups indicate $p \leq 5e^{-2}$.

Supplementary Information 2.8, pp. 13

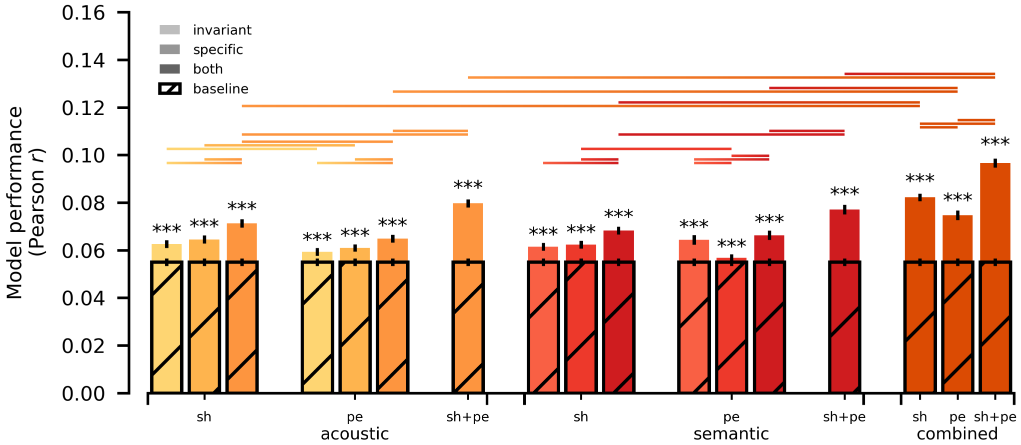

Supplementary Fig. 8 Between-item RSA regression models, setting $k = 1$ to avoid averaging and restricting the temporal window of the analysis to the shortest possible prediction (0.0-0.48s) such that differences in length could not affect results (see Conventional representational similarity analysis). Results corroborate the pattern observed without temporal restrictions (see Supplementary Fig. 7), ruling out prediction length as a confounding factor (see Supplementary Table 8). Individual bars represent group means, with black lines indicating 95%-confidence intervals around the mean. ***, **, * indicate $p \leq 1e^{-3}$, $p \leq 1e^{-2}$, and $p \leq 5e^{-2}$, respectively. Big bars between groups indicate $p \leq 5e^{-2}$.

3. Granularity of tests

The discrepancy between acoustic sharpening and acoustic prediction error findings might arise from different analysis granularities. Sharpening is assessed with representation-content measures, while acoustic prediction error is tested using a univariate cosine-distance metric.

Thank you for raising this important point. We fully agree that the granularity of tests may affect the results, and believe this to be one of the core findings of the paper. However, in within-item cRSA, we did not unilaterally test for sharpening. Instead, we contrasted sharpening and prediction error computations directly. While we found sharpening to be the dominant computation at the sensory level, we could have equally found prediction error representations here. In fact, results from a complementary conventional RSA presented above suggest that there is potential for sharpening and prediction error to co-occur at the sensory level (see Conventional representational similarity analysis, Supplementary Figs. 7-8). This, however, stands in contrast to results from the following encoding models where, for comparibility with prior work[30,31], we estimate prediction error coefficients from univariate surprisal metrics.

We now address this intriguing discrepancy more explicitly in the discussion. Please also the changes to the discussion following comment 1 on page 44. Beyond that, we have made the following changes to the manuscript:

Discussion, pp. 16, lines 453+

[...]

Yet, while these supplementary RSA results hint at weakly co-occurring acoustic prediction error at the sensory level (see Supplementary Figs. 7-8), we do not find evidence thereof in regressions of sensory cRSMs (see Fig. 2d) or encoding models of broadband EEG responses (see Fig. 3). In the case of cRSM regression, this is expected given that supplementary RSA results show acoustic sharpening to be considerably stronger than acoustic prediction errors (see Supplementary Fig. 7): When investigating the directionality of the representational shift overall, the stronger sharpening effect should dominate the weaker prediction error effect. That encoding models of broadband EEG responses do not support acoustic prediction errors (see Fig. 3d; Fig. 4) demonstrates the critical role that analysis granularity may play. As with sharpening, it is possible that multivariate sensory prediction errors are not easily detectable in commonly employed broadband encoding of univariate distance metrics[30,31]. In all, investigating predictive processing in natural speech by incorporating a wider array of methodologies–particularly, placing greater importance on representational content, laminar resolution of activity, and task-demands– may allow comparing and disentangling sharpening and prediction error computations at the acoustic level across monologic and dialogic contexts more thoroughly. Within regions, sharpening and prediction errors may be represented in deep and superficial layers, respectively[3,4], and across regions sharpening may dominate prediction errors at the sensory level[5] unless task-demands place greater importance on deviation detection[2].

4. Statistical analyses

I am concerned by the extremely low p-values (e.g., $p < 10^{-19}$ for RTs). Such values often indicate mis-specified models, pseudo-replication, or overfitting. Looking at the LMER model specification, they look quite unusual to me. E.g. the model from p. 28:

$$\begin{aligned}&(1 + t + p(\text{word}|\text{speaker})|\text{participant} \times \text{context})\\&+ (1|\text{participant} \times \text{position})\\&+ (1|\text{participant} \times \text{stimulus})\\&+ (1|\text{face} \times \text{feature})\end{aligned}$$

is unconventional. The inclusion of random variables such as participants:stimulus indicates that there is a separate level for *each combination of item and participant*.

I presume 'x' in tje models means '*' in lmer specification, i.e. full factorial design. I would expect that such models will not converge, and at the very least, it would be useful to remove random correlations.

I think the formulation should look more like:

$$
\begin{aligned}
lmer(Y \sim & \text{ predictors}+ \\
& + (1 + \text{applicable\_predictors}||\text{participants}) \\
& + (1 + \text{applicable\_predictors}||\text{context})\# \text{ I was unsure what context refers to} \\
& + (1 + \text{applicable\_predictors}||\text{stimulus})
\end{aligned}
$$

We thank the reviewer for pointing out the potential for pseudoreplication here. We have confirmed that all results hold after refitting the maximal model following their suggestions. Additionally, we also verified GLMER models were unaffected by this. We have updated the corresponding figure (see Fig 4b) following reanalysis and have made the following changes to the manuscript:

Methods 4.13, pp. 32, lines 1038+

Modeling behaviour in congruent and incongruent trials

Reaction times were analysed in R (version 4.03)[61] using lme4[62] and emmeans[63]. Linear mixed models were fit with default parameters. A maximal modelling procedure was followed[64]. Residuals of the identified maximal model were inspected visually.

Reaction times were modeled as

$$\log \text{RT} \sim t \times \text{congruence} \times p(\text{word}|\text{speaker}) + (1|\text{participant})$$

where trial number t and the probability of the word given the speaker $p(\text{word}|\text{speaker})$ were scaled to have zero mean and unit variance. For the random effects structure, we included all reasonable permutations of these predictors as well as the position of the word on screen, the semantic context, the speaker and their distinguishing visual feature. The maximal identifiable model included:

$$
\begin{aligned}
&(1 + t + p(\text{word}|\text{speaker})||\text{participant}) \\
&+ (1 + p(\text{word}|\text{speaker})||\text{context}) \\
&+ (1 + p(\text{word}|\text{speaker})||\text{stimulus}) \\
&+ (1 + p(\text{word}|\text{speaker})||\text{position})
\end{aligned}
$$

Results 2.4, pp. 13, lines 352+

Participants were significantly slower in incongruent trials, suggesting increased surprisal or decisional conflict (linear mixed model (LMM), $\beta = 0.10, s.e. = 0.02, t(1606) = 5.41, p = 7.30e^{-8}$, see Fig. 4b; see also Modeling behaviour in congruent and incongruent trials). Crucially, reaction times scaled with the probability of a word given the speaker in congruent trials (estimated marginal trends (EMT) at $\mathbb{E}[p(\text{word}|\text{speaker})]$, $\beta = -0.12, s.e. = 0.02, t(9) = -5.53, p = 3.33e^{-4}$), but not in incongruent trials (EMT, $\beta = -0.02, s.e. = 0.02, t(14) = -0.89, p = 0.39$; see Fig. 4b). For incongruent trials, this effect emerged only over trials (LMM, congruence $\times$ *trial* $\times$ p(word|speaker): $\beta = -0.03, s.e. = 0.02, t(3976) = -2.07, p = 3.83e^{-2}$). Thus, while participants continued to leverage speaker-specific priors, they seemed to discard them initially when encountering highly improbable words, incurring a switch cost.

Minor comments

1) p.6 - "Firstly, we built acoustic hypothesis RSMs from spectrograms of clear words that were predictable from participants' speaker-specific and -invariant semantic priors."
- How can you know which words were expected to participants when discarding information about the speaker?
- How do you know which category-specific words were the most predictable knowing the speaker? There are probably dozens of exemplars fitting a semantic category. Also, before the experiment, participants don't know which 20 exemplars will be used.

We thank the reviewer for highlighting this concern. We have made the following changes to address this:

Supplementary Methods 1.1, pp. 1

Our experimental design was tailored to address the question: How do expectations shape neural representations? Principally, a morph *sea*-*tea* given speaker context *nature* may be represented more like *sea* than *tea*–or vice versa. However, it is unlikely that the brain makes predictions that are this concrete, as it would require, for example, predicting *sea* at trial t (*sea*-*tea*|*nature*) but immediately thereafter *kayak* at trial $t+1$ (*kayak*-*cognac*|*nature*). In reality, the brain is assumed to make distributional predictions instead[24]. For

example, the distributional expectation for *nature* $q(nature)$ may be:

$$q(nature) = \begin{bmatrix} p(sea|nature) \\ p(kayak|nature) \\ \vdots \\ p(tea|nature) \end{bmatrix} = \begin{bmatrix} 0.14 \\ 0.10 \\ \vdots \\ 0.02 \end{bmatrix} \tag{3}$$

where $p(word_i|context)$ is computed from semantic embeddings of individual words e_i and semantic priors obtained from free-energy models π:

$$p(w_i|\pi) = \frac{1}{2\bar{p}} \left(\frac{e_i \cdot \pi}{||e_i||\ ||\pi||} + 1 \right) \qquad \text{where} \qquad \bar{p} = \sum_{i=0}^{k} p(w_i|\pi)$$

In other words, the probability of any word given a context is the normalised cosine similarity $\tilde{\angle} \in [0, 1]$ between the semantic embedding of that word and the semantic embedding of the context obtained from free-energy models that evolve over the experiment, measuring their probability as a function of how similar a word is to a given prior. Note that, following prior work[48], we use cosine similarity rather than the likelihood of e_i under $\mathcal{N}(\mu_\pi, \sigma_\pi^2)$ as a computational simplification that works particularly well in high-dimensional semantic embeddings. Note also that context subsumes not only speaker-specific priors, but also speaker-invariant priors (i.e., context across the experiment).

Generally, we may then obtain an estimate of the prediction as:

$$\mathbb{E}_f(\pi) = \sum_{i=0}^{N} p(w_i|\pi) f(w_i) \qquad \text{where} \qquad \sum_{i=0}^{N} p(w_i|\pi) = 1$$

where f describes some feature we are interested in (for example, acoustic spectrograms), π refers to the speaker-specific or speaker-invariant semantic prior we are applying, and w_i is word i of N total words.

While this simplifies the prediction problem by assuming only a fixed vocabulary over stimuli included in the study, we can be reasonably certain that these truly reflect expected words. This is because, during stimulus creation, categories in semantic space were sampled densely, including those words that most closely aligned with any category. Therefore, these words should inherently reflect the most predictable words given any context. [...]

2) p.6 - "Secondly, we generated semantic hypothesis RSMs by comparing the semantic embeddings of the congruent words with the current estimates

of the speaker-specific and speaker-invariant semantic priors (see Fig. 2b)"
- What were the congruent words? I assume you are not referring here to the next procedure in which you used congruent and incongruent words, but rather mean the speaker-congruent part of the morphed word?
- I assume speaker-specific priors are computed using the free-energy optimization? if yes, an explicit link to that section of the paper would be useful

Many thanks for catching this. We have now clarified this:

Results 2.2, pp. 7, lines 183+

Secondly, we generated semantic hypothesis cRSMs by comparing the semantic embeddings of the constituent words (e.g., *sea*, *tea* for morph *sea-tea*) with the current estimates of the speaker-specific and speaker-invariant semantic priors from free-energy models (see Fig. 2b, see also Estimating real-time semantic models).

3) p. 6 - "Encoding models" is misleading; in neuroscience, this usually means predicting brain activity from stimuli, not regressing RSMs on each other. It would be clearer of the TRF analysis was called TRF.

Thank you for pointing out the mismatch in nomenclature here. As seen in general comments #1 and #2, we now consistently refer to this as regression in the case of cRSA. Further, we have now clarified the use of TRFs:

Results 2.3, pp. 11, lines 268+

Building on our finding that speaker-specific semantic priors sharpen low-level sensory representations, we next examined whether prediction errors emerge at higher levels of the speech-processing hierarchy instead. To do this, we built encoding models (i.e., multivariate temporal response functions[15–17], mTRFs) of single-trial EEG signals that allowed us to probe signatures of acoustic and semantic prediction errors in the broadband EEG response. [...]

Methods 4.11, pp. 30, lines 967+

To investigate whether participants showed neural signatures of speaker-specific acoustic or semantic surprisal, we built encoding models[15–17] mapping from stimulus features X to the recorded neural data y through multivariate temporal response functions (mTRFs) for each participant: [...]

4) p. 11 - Since you mention EMTs, at what reference values they were computed?

EMTs were computed at the expected value. Given that predictors were z-scored, $\mathbb{E}[p(\text{word}|\text{speaker})] = 0$. This was done because from the base LMM,

we could only infer that congruency impacted the degree to which response latencies scaled with the probability of the word under the speaker prior. However, we could not infer whether or not this scaling was apparent in both congruency conditions. Therefore, we tested EMTs at 0. We have clarified this in the manuscript:

Results 2.4, pp. 13, lines 352+

Participants were significantly slower in incongruent trials, suggesting increased surprisal or decisional conflict (linear mixed model (LMM), $\beta = 0.10, s.e. = 0.02, t(1606) = 5.41, p = 7.30e^{-8}$, see Fig. 4b; see also Modeling behaviour in congruent and incongruent trials). Crucially, reaction times scaled with the probability of a word given the speaker in congruent trials (estimated marginal trends (EMT) at $\mathbb{E}\left[p(\text{word}|\text{speaker})\right]$, $\beta = -0.12, s.e. = 0.02, t(9) = -5.53, p = 3.33e^{-4}$), but not in incongruent trials (EMT, $\beta = -0.02, s.e. = 0.02, t(14) = -0.89, p = 0.39$; see Fig. 4b). For incongruent trials, this effect emerged only over trials (LMM, congruence $\times$ *trial* $\times$ $p(\text{word}|\text{speaker})$: $\beta = -0.03, s.e. = 0.02, t(3976) = -2.07, p = 3.83e^{-2}$). Thus, while participants continued to leverage speaker-specific priors, they seemed to discard them initially when encountering highly improbable words, incurring a switch cost.

5) p.23 - What was the control predictor? Please explain its purpose, what were the sampled priors?

Here, control predictors were computed because language in general is highly predictable. Consequently, even without speaker-specific or -invariant priors, we would expect to find general predictions of spectral or semantic features. Therefore, control predictors were included that mimicked this general predictability to ensure that speaker-invariant and -specific predictions each explained additional unique variance. Control predictors followed the exact same computations as hypothesis predictors, except that we generated them from non-specific semantic priors that were samples of a Gaussian in semantic space at each trial. For a large numbers of trials and k, this should approximate more general acoustic or semantic expectations that were not tied to our experiment but reflect statistical regularities of German instead. We have clarified this in the manuscript:

Methods 4.9, pp. 27, lines 869+

Because language exhibits strong baseline predictability[48,65], we controlled for general acoustic expectations that participants may have about German. To do this, we repeated the exact procedure for top-k predictions outlined above, but generated predictions from non-specific priors sampled from an isotropic Gaussian around the

vector space's centroid at every trial. Across large numbers of trials and k, this therefore approximates general statistical regularities of German, allowing us to control for general predictive processes that were irrespective of our experiment.

6) p. 23 and elsewhere - p(speaker) implies the probability of a specific speaker. But after careful reading one realizes that the authors mean instead a semantic prior distribution from the free-energy model. Possibly names of other variables in formula could also be clarified.

We thank the reviewer for highlighting this lack of clarity. In addition to prior changes to names, we have made the following changes to the manuscript:

Methods 4.9, pp. 27, lines 877+

Prior-dependent semantic representation. If semantic priors influence the processing of the morphed words at higher-level processing stages—for example, shifting sensory representations uniformly per semantic context—composite representational similarity should reflect direct comparisons between the prior distribution of the semantics given the speaker π(speaker) derived from free-energy models (see Estimating real-time semantic models) and the word embeddings of the words making up the morph (e.g., *sea* and *tea*). As before, we computed an additional control predictor to capture more global statistical regularities.

7) p. 26-27 - The "semantic prediction error" control variable (cosine distance from GloVe embedding to mean embedding of all words) seems ill-motivated. If the lexicon's centroid is near zero, the angle is unstable and not interpretable.

We thank the reviewer for bringing forth this concern. However, if angles were too unstable, we would expect not to find consistent variance uniquely attributable to general semantic surprisal in back-to-back decoding from wav2vec2.0 activations (see Supplementary Fig. 9).

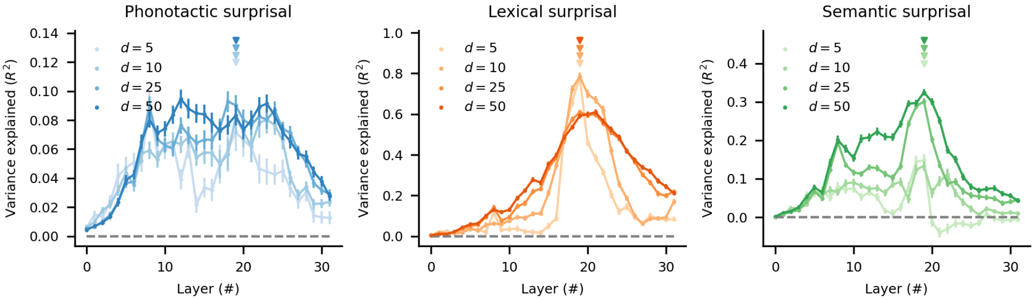

Supplementary Fig. 9 To identify the layer within word2vec2.0 that best captured phonotactic, lexical and semantic surprisal, we performed back-to-back decoding[21] over an independent set of narrative stimuli. Here, we show the relative causal contribution of each feature across layers, for a number of PCA projections with dimensionality d. Small dots represent means, with 95%-confidence intervals around them. Downward-facing triangles represent the best layer overall for each d. Crucially, for all d tested, layer 19 (transformer layer 12) was identified as the most informative layer. This is in accordance with recent results finding that middle layers of LLMs tend to reflect neural processing best[65,66].

8) p.26 - "Note that coefficients w(t, n) in these models are not strictly temporal response functions given that we used standardised (i.e., dense) design matrices which allowed us to model non-linearly additive effects of consecutive events, at the cost of interpreting coefficients in reference to baseline levels rather than as discrete events."
- It is unclear to me what the authors mean by "standardised (i.e. dense) design matrices", and how it implies nonlinearity. If the continuous nature of wav2vec is a problem, you could always average wav2vec embeddings within the analysed unit (phonemes or words) to convert them to discrete events, i.e. spike regressors. But maybe I misunderstood the problem.

Many thanks for pointing us to this confusing wording which wrongly implicated that we employed some kind of non-linear linking function. We now clarify that continuous predictors had already been converted to spike regressors:

Methods 4.11, pp. 30, lines 966+

Encoding of single-trial EEG data

To investigate whether participants showed neural signatures of speaker-specific acoustic or semantic surprisal, we built encoding models[15–17] mapping from stimulus features X to the recorded neural data y through multivariate temporal response functions (mTRFs) for each participant:

$$r(t, n) = \sum_{\tau} w(\tau, n) s(t - \tau) + \epsilon(t, n)$$

> Here $r(t, n)$ is the reconstructed neural signal in channel n at time point t, $w(\tau, n)$ are the β-estimates at lag τ, $s(t-\tau)$ are the corresponding stimulus features, and $\epsilon(t, n)$ are the residuals. For τ, we chose a window between $-100ms$ and $800ms$. Continuous predictors (e.g., acoustic envelopes, wav2vec2.0 activations) were averaged within each linguistic unit to create discrete predictors at the phoneme and word levels. For features without explicit segmentation (e.g., wav2vec2.0 activations), we constructed both phoneme- and word-level spike regressors in this way. We solved for the forward model W using sklearn[52] and PyTorch[50]:
>
> $$\arg\min_W \sum_t (y_t - W^T S_t)^2 + \alpha_W ||W||^2$$
>
> where optimal ridge penalties α_W were found using leave-one-out cross-validation to test 20 logarithmically spaced values between $1e^{-5} - 1e^{10}$. All models were estimated using 5-fold cross-validation with 50 repetitions[55] and all outcomes and predictors were scaled to have zero mean and unit variance based on the training set to prevent data leakage. For predictors, standardisation was applied after lag expansion (i.e., each column of the lagged design matrix S_t was z-scored within the training fold) to ensure that ridge regularisation penalised all lags comparably. Out-of-sample performance was defined as the Pearson correlation between observed neural signals $y(t, n)$ and reconstructions thereof $r(t, n)$ in the held-out test set[15].

9) p. 27 - Could you report how much variance was explained by the baseline models and how it improved by adding acoustic and semantic surprisal measures?

Thanks for highlighting the missing baseline reporting. Baseline performance was already relatively high (Pearson $r = 0.18$ and $r = 0.15$ in main and exemplar tasks, respectively). Consequently, relative gains of individual models (see Fig. 3b, 4c) were very modest but highly consistent across participants, though this is not uncommon in mTRF analyses with a high number of predictors[22]. We have made the following changes to the manuscript:

> **Methods 4.11, pp. 31, lines 1009+**
>
> **Statistical inference.** Encoding performance improvements were tested using two-sided paired t-tests, with Bonferroni-Holm corrections applied for multiple comparisons. Specifically, we tested for within-participant differences between models, e.g. t(baseline, speaker-specific acoustic). Encoding performance of baseline models–measured as the Pearson correlation between observed neural responses y and reconstructions thereof r–was confirmed to be above

chance using one-sample t-tests (main task: $M = 0.18$, $s.e. = 0.01$, $t(34) = 26.48, p = 2.80e^{-24}$; exemplar task: $M = 0.15$, $s.e. = 0.01$, $t(34) = 20.92, p = 5.36^{-21}$).

10) p. 27 - Statistical inference - what exactly was compared in the t-test (and in other places where models are compared)?

Thank you for highlighting the lack of clarity here. We have clarified the kinds of model comparisons throughout the manuscript. Generally, we compared within-participant model performance using paired samples t-tests of the form t(model_0, model_1).

11) p.27 - "Baseline models also included either projected LLM activations or direct measures of phonotactic, lexical and semantic surprisal, relative to the target word" - I'm confused by the word "or".

The fundamental problem we faced when modeling mTRFs to morphed words, is that lexical and semantic surprisal are technically undefined for morphs, as computing surprisal would require disambiguating the morph. Our aim was to control for general acoustic, lexical and semantic surprisal that are known to modulate the neural response. We describe these details in *2.3 Speaker-specific semantic priors induce prediction error representations at higher levels* and *4.10 Pretrained transformers as statistical surrogates.*

We addressed this issue by using wav2vec2.0 activations as a surrogate measure of surprisal after confirming all acoustic, lexical, and semantic surprisal were uniquely decodeable from projected activations (see Supplementary Fig. 9). Yet, it was conceivable that wav2vec2.0 would struggle with morphs itself, potentially biassing results. Therefore, we chose to run separate complementary analyses where we did not use wav2vec2.0 activations to control for these but computed surprisal values directly by disambiguating the morph towards the target word.

We clarified this in the manuscript:

Methods 4.11, pp. 31, lines 994+

[...] Baseline models also included predictors controlling for general phonotactic, lexical and semantic surprisal. Generally, control predictors were derived from pretrained transformer activations (see Pretrained transformers as statistical surrogates). However, to rule out the possibility of performance of pretrained transformers given morphs confounding results, we also conducted a second set of analyses where control predictors were computed as direct measures of phonotactic, lexical and semantic surprisal by disambiguating the morph to the target word.

12) I think the authors could cite Broderick et al., 2019 - who also showed results consistent with acoustic sharpening in auditory speech comprehension.

Many thanks for catching this missing reference. We now cite this paper in the manuscript's introduction (and at several places in the discussion).

References

[1] Garlichs, A., Blank, H.: Prediction error processing and sharpening of expected information across the face-processing hierarchy. Nat Commun **15**(1), 3407 (2024). https://doi.org/10.1038/s41467-024-47749-9. Accessed 2025-01-16

[2] Alink, A., Blank, H.: Can expectation suppression be explained by reduced attention to predictable stimuli? NeuroImage **231**, 117824 (2021). https://doi.org/10.1016/j.neuroimage.2021.117824. Accessed 2025-02-18

[3] Aitken, F., Menelaou, G., Warrington, O., Koolschijn, R.S., Corbin, N., Callaghan, M.F., Kok, P.: Prior expectations evoke stimulus-specific activity in the deep layers of the primary visual cortex. PLoS Biol **18**(12), 3001023 (2020). https://doi.org/10.1371/journal.pbio.3001023. Accessed 2025-09-19

[4] Thomas, E.R., Haarsma, J., Nicholson, J., Yon, D., Kok, P., Press, C.: Predictions and errors are distinctly represented across V1 layers. Current Biology **34**(10), 2265–22714 (2024). https://doi.org/10.1016/j.cub.2024.04.036. Accessed 2025-09-19

[5] Press, C., Kok, P., Yon, D.: The Perceptual Prediction Paradox. Trends in Cognitive Sciences **24**(1), 13–24 (2020). https://doi.org/10.1016/j.tics.2019.11.003. Accessed 2023-11-15

[6] Kriegeskorte, N.: Representational similarity analysis – connecting the branches of systems neuroscience. Front. Sys. Neurosci. (2008). https://doi.org/10.3389/neuro.06.004.2008. Accessed 2025-01-22

[7] Aitchison, L., Lengyel, M.: With or without you: predictive coding and Bayesian inference in the brain. Current Opinion in Neurobiology **46**, 219–227 (2017). https://doi.org/10.1016/j.conb.2017.08.010. Accessed 2022-10-11

[8] Spratling, M.W.: A review of predictive coding algorithms. Brain and Cognition **112**, 92–97 (2017). https://doi.org/10.1016/j.bandc.2015.11.003. Accessed 2025-09-08

[9] Heeger, D.J.: Theory of cortical function. Proc. Natl. Acad. Sci. U.S.A. **114**(8), 1773–1782 (2017). https://doi.org/10.1073/pnas.1619788114. Accessed 2025-09-08

[10] Millidge, B., Seth, A., Buckley, C.L.: Predictive Coding: a Theoretical and Experimental Review (2021). https://doi.org/10.48550/ARXIV.2107.12979. Accessed 2023-11-13

[11] Lian, Y., Burkitt, A.N.: Relating sparse and predictive coding to divisive normalization. PLoS Comput Biol **21**(5), 1013059 (2025). https://doi.org/10.1371/journal.pcbi.1013059. Accessed 2025-09-08

[12] Bowers, J.S., Davis, C.J.: Bayesian just-so stories in psychology and neuroscience. Psychological Bulletin **138**(3), 389–414 (2012). https://doi.org/10.1037/a0026450. Accessed 2025-09-08

[13] Miłkowski, M., Litwin, P.: Testable or bust: theoretical lessons for predictive processing. Synthese **200**(6), 462 (2022). https://doi.org/10.1007/s11229-022-03891-9. Accessed 2025-09-08

[14] Bowman, H., Collins, D.J., Nayak, A.K., Cruse, D.: Is predictive coding falsifiable? Neuroscience & Biobehavioral Reviews **154**, 105404 (2023). https://doi.org/10.1016/j.neubiorev.2023.105404. Accessed 2025-09-08

[15] Crosse, M.J., Di Liberto, G.M., Bednar, A., Lalor, E.C.: The Multivariate Temporal Response Function (mTRF) Toolbox: A MATLAB Toolbox for Relating Neural Signals to Continuous Stimuli. Front. Hum. Neurosci. **10** (2016). https://doi.org/10.3389/fnhum.2016.00604. Accessed 2024-01-19

[16] Smith, N.J., Kutas, M.: Regression-based estimation of ERP waveforms: I . The rERP framework. Psychophysiology **52**(2), 157–168 (2015). https://doi.org/10.1111/psyp.12317. Accessed 2024-01-23

[17] Smith, N.J., Kutas, M.: Regression-based estimation of ERP waveforms: II . Nonlinear effects, overlap correction, and practical considerations. Psychophysiology **52**(2), 169–181 (2015). https://doi.org/10.1111/psyp.12320. Accessed 2024-11-25

[18] Baevski, A., Zhou, H., Mohamed, A., Auli, M.: wav2vec 2.0: A Framework for Self-Supervised Learning of Speech Representations. arXiv (2020). https://doi.org/10.48550/ARXIV.2006.11477. https://arxiv.org/abs/2006.11477 Accessed 2025-01-22

[19] Anderson, A.J., Davis, C., Lalor, E.C.: Deep-learning models reveal how context and listener attention shape electrophysiological correlates of speech-to-language transformation. PLoS Comput Biol **20**(11), 1012537 (2024). https://doi.org/10.1371/journal.pcbi.1012537. Accessed 2025-03-26

[20] Goldstein, A., Grinstein-Dabush, A., Schain, M., Wang, H., Hong, Z., Aubrey, B., Nastase, S.A., Zada, Z., Ham, E., Feder, A., Gazula, H.,

Buchnik, E., Doyle, W., Devore, S., Dugan, P., Reichart, R., Friedman, D., Brenner, M., Hassidim, A., Devinsky, O., Flinker, A., Hasson, U.: Alignment of brain embeddings and artificial contextual embeddings in natural language points to common geometric patterns. Nat Commun **15**(1), 2768 (2024). https://doi.org/10.1038/s41467-024-46631-y. Accessed 2025-03-26

[21] King, J.-R., Charton, F., Lopez-Paz, D., Oquab, M.: Back-to-back regression: Disentangling the influence of correlated factors from multivariate observations. NeuroImage **220**, 117028 (2020). https://doi.org/10.1016/j.neuroimage.2020.117028. Accessed 2025-01-22

[22] Brodbeck, C., Das, P., Gillis, M., Kulasingham, J.P., Bhattasali, S., Gaston, P., Resnik, P., Simon, J.Z.: Eelbrain, a Python toolkit for time-continuous analysis with temporal response functions. eLife **12**, 85012 (2023). https://doi.org/10.7554/eLife.85012. Accessed 2025-09-11

[23] de Lange, F.P., Heilbron, M., Kok, P.: How Do Expectations Shape Perception? Trends in Cognitive Sciences **22**(9), 764–779 (2018). https://doi.org/10.1016/j.tics.2018.06.002. Accessed 2022-10-11

[24] Friston, K.: The free-energy principle: a unified brain theory? Nat Rev Neurosci **11**(2), 127–138 (2010). https://doi.org/10.1038/nrn2787. Accessed 2024-01-23

[25] Broderick, M.P., Anderson, A.J., Lalor, E.C.: Semantic Context Enhances the Early Auditory Encoding of Natural Speech. J. Neurosci. **39**(38), 7564–7575 (2019). https://doi.org/10.1523/JNEUROSCI.0584-19.2019. Accessed 2025-02-12

[26] Corcoran, A.W., Perera, R., Koroma, M., Kouider, S., Hohwy, J., Andrillon, T.: Expectations boost the reconstruction of auditory features from electrophysiological responses to noisy speech. Cerebral Cortex **33**(3), 691–708 (2023). https://doi.org/10.1093/cercor/bhac094. Accessed 2025-05-26

[27] Rothermich, K., Kotz, S.A.: Predictions in speech comprehension: fMRI evidence on the meter–semantic interface. NeuroImage **70**, 89–100 (2013). https://doi.org/10.1016/j.neuroimage.2012.12.013. Accessed 2025-09-19

[28] Blank, H., Davis, M.H.: Prediction Errors but Not Sharpened Signals Simulate Multivoxel fMRI Patterns during Speech Perception. PLoS Biol **14**(11), 1002577 (2016). https://doi.org/10.1371/journal.pbio.1002577. Accessed 2023-06-28

[29] Blank, H., Spangenberg, M., Davis, M.H.: Neural Prediction Errors Distinguish Perception and Misperception of Speech. J. Neurosci. **38**(27),

6076–6089 (2018). https://doi.org/10.1523/JNEUROSCI.3258-17.2018. Accessed 2025-02-13

[30] Donhauser, P.W., Baillet, S.: Two Distinct Neural Timescales for Predictive Speech Processing. Neuron **105**(2), 385–3939 (2020). https://doi.org/10.1016/j.neuron.2019.10.019. Accessed 2025-09-08

[31] Heilbron, M., Armeni, K., Schoffelen, J.-M., Hagoort, P., De Lange, F.P.: A hierarchy of linguistic predictions during natural language comprehension. Proc. Natl. Acad. Sci. U.S.A. **119**(32), 2201968119 (2022). https://doi.org/10.1073/pnas.2201968119. Accessed 2023-06-12

[32] Sohoglu, E., Beckers, L., Davis, M.H.: Convergent neural signatures of speech prediction error are a biological marker for spoken word recognition. Nat Commun **15**(1), 9984 (2024). https://doi.org/10.1038/s41467-024-53782-5. Accessed 2025-09-19

[33] Kok, P., Jehee, J.M., de Lange, F.: Less Is More: Expectation Sharpens Representations in the Primary Visual Cortex. Neuron **75**(2), 265–270 (2012). https://doi.org/10.1016/j.neuron.2012.04.034. Accessed 2024-12-18

[34] Cui, L., Bo, K., Xiong, C., Chen, Y., Keil, A., Ding, M.: Stimulus Repetition Induces a Two-Stage Learning Process in the Primary Visual Cortex. J. Neurosci. **45**(23), 1788242025 (2025). https://doi.org/10.1523/JNEUROSCI.1788-24.2025. Accessed 2025-09-19

[35] Richter, D., Heilbron, M., De Lange, F.P.: Dampened sensory representations for expected input across the ventral visual stream. Oxford Open Neuroscience **1**, 013 (2022). https://doi.org/10.1093/oons/kvac013. Accessed 2025-09-19

[36] Richter, D., Kietzmann, T.C., De Lange, F.P.: High-level visual prediction errors in early visual cortex. PLoS Biol **22**(11), 3002829 (2024). https://doi.org/10.1371/journal.pbio.3002829. Accessed 2025-09-19

[37] Jaramillo, S., Zador, A.: Auditory cortex mediates the perceptual effects of acoustic temporal expectation. Nat Prec (2010). https://doi.org/10.1038/npre.2010.5139.1. Accessed 2025-02-05

[38] Kuperberg, G.R., Brothers, T., Wlotko, E.W.: A Tale of Two Positivities and the N400: Distinct Neural Signatures Are Evoked by Confirmed and Violated Predictions at Different Levels of Representation. Journal of Cognitive Neuroscience **32**(1), 12–35 (2020). https://doi.org/10.1162/jocn_a_01465. Accessed 2024-01-22

[39] Samuelsson, J.G., Peled, N., Mamashli, F., Ahveninen, J., Hämäläinen,

M.S.: Spatial fidelity of MEG/EEG source estimates: A general evaluation approach. NeuroImage **224**, 117430 (2021). https://doi.org/10.1016/j.neuroimage.2020.117430. Accessed 2025-10-07

[40] Halder, T., Talwar, S., Jaiswal, A.K., Banerjee, A.: Quantitative Evaluation in Estimating Sources Underlying Brain Oscillations Using Current Source Density Methods and Beamformer Approaches. eNeuro **6**(4), 0170–192019 (2019). https://doi.org/10.1523/ENEURO.0170-19.2019. Accessed 2025-10-07

[41] Draganov, M., Galiano-Landeira, J., Doruk Camsari, D., Ramírez, J.-E., Robles, M., Chanes, L.: Noninvasive modulation of predictive coding in humans: causal evidence for frequency-specific temporal dynamics. Cerebral Cortex, 127 (2023). https://doi.org/10.1093/cercor/bhad127. Accessed 2023-06-12

[42] Aizenbud, I., Audette, N., Auksztulewicz, R., Basiński, K., Bastos, A.M., Berry, M., Canales-Johnson, A., Choi, H., Clopath, C., Cohen, U., Costa, R.P., De Filippo, R., Doronin, R., Errington, S.P., Gavornik, J.P., Gillon, C.J., Granier, A., Hamm, J.P., Hertäg, L., Kennedy, H., Kumar, S., Ladd, A., Ladret, H., Lecoq, J.A., Maier, A., McCarthy, P., Mei, J., Mejias, J., Mikulasch, F., Mudrik, N., Najafi, F., Nejad, K., Nejat, H., Oweiss, K., Petrovici, M.A., Priesemann, V., Rudelt, L., Ruediger, S., Russo, S., Salatiello, A., Senn, W., Sennesh, E., Sima, S., Uran, C., Vasilevskaya, A., Vezoli, J., Vinck, M., Westerberg, J.A., Wilmes, K., Xiong, Y.S.: Neural mechanisms of predictive processing: a collaborative community experiment through the OpenScope program. arXiv. Version Number: 1 (2025). https://doi.org/10.48550/ARXIV.2504.09614. https://arxiv.org/abs/2504.09614 Accessed 2025-10-07

[43] Hickok, G., Poeppel, D.: The cortical organization of speech processing. Nat Rev Neurosci **8**(5), 393–402 (2007). https://doi.org/10.1038/nrn2113. Accessed 2025-10-07

[44] Kutas, M., Federmeier, K.D.: Thirty Years and Counting: Finding Meaning in the N400 Component of the Event-Related Brain Potential (ERP). Annu. Rev. Psychol. **62**(1), 621–647 (2011). https://doi.org/10.1146/annurev.psych.093008.131123. Accessed 2025-10-07

[45] Lau, E.F., Phillips, C., Poeppel, D.: A cortical network for semantics: (de)constructing the N400. Nat Rev Neurosci **9**(12), 920–933 (2008). https://doi.org/10.1038/nrn2532. Accessed 2025-02-12

[46] Meyer, S.S., Bonaiuto, J., Lim, M., Rossiter, H., Waters, S., Bradbury, D., Bestmann, S., Brookes, M., Callaghan, M.F., Weiskopf, N., Barnes, G.R.: Flexible head-casts for high spatial precision MEG. Journal of Neuroscience Methods **276**, 38–45 (2017). https://doi.org/10.1016/j.jneumeth.

2016.11.009. Accessed 2025-10-07

[47] Bonaiuto, J.J., Rossiter, H.E., Meyer, S.S., Adams, N., Little, S., Callaghan, M.F., Dick, F., Bestmann, S., Barnes, G.R.: Non-invasive laminar inference with MEG: Comparison of methods and source inversion algorithms. NeuroImage **167**, 372–383 (2018). https://doi.org/10.1016/j.neuroimage.2017.11.068. Accessed 2025-10-07

[48] Heilbron, M., Chait, M.: Great Expectations: Is there Evidence for Predictive Coding in Auditory Cortex? Neuroscience **389**, 54–73 (2018). https://doi.org/10.1016/j.neuroscience.2017.07.061. Accessed 2022-10-11

[49] Schneider, F.: predcommlab/hearing_is_believing. predcommlab. original-date: 2025-02-17T13:35:07Z (2025). https://github.com/predcommlab/hearing_is_believing Accessed 2025-10-07

[50] Paszke, A., Gross, S., Massa, F., Lerer, A., Bradbury, J., Chanan, G., Killeen, T., Lin, Z., Gimelshein, N., Antiga, L., Desmaison, A., Köpf, A., Yang, E., DeVito, Z., Raison, M., Tejani, A., Chilamkurthy, S., Steiner, B., Fang, L., Bai, J., Chintala, S.: PyTorch: An Imperative Style, High-Performance Deep Learning Library (2019). https://doi.org/10.48550/ARXIV.1912.01703. Accessed 2023-06-29

[51] Schneider, F.: FabulousFabs/MVPy. original-date: 2025-02-25T08:02:50Z (2025). https://github.com/FabulousFabs/MVPy Accessed 2025-09-10

[52] Pedregosa, F., Varoquaux, G., Gramfort, A., Michel, V., Thirion, B., Grisel, O., Blondel, M., Müller, A., Nothman, J., Louppe, G., Prettenhofer, P., Weiss, R., Dubourg, V., Vanderplas, J., Passos, A., Cournapeau, D., Brucher, M., Perrot, M., Duchesnay, : Scikit-learn: Machine Learning in Python (2012). https://doi.org/10.48550/ARXIV.1201.0490. Accessed 2025-01-22

[53] facebook/wav2vec2-large-xlsr-53-german · Hugging Face (2024). https://huggingface.co/facebook/wav2vec2-large-xlsr-53-german Accessed 2025-01-22

[54] Wolf, T., Debut, L., Sanh, V., Chaumond, J., Delangue, C., Moi, A., Cistac, P., Rault, T., Louf, R., Funtowicz, M., Davison, J., Shleifer, S., von Platen, P., Ma, C., Jernite, Y., Plu, J., Xu, C., Scao, T.L., Gugger, S., Drame, M., Lhoest, Q., Rush, A.M.: HuggingFace's Transformers: State-of-the-art Natural Language Processing (2019). https://doi.org/10.48550/ARXIV.1910.03771. Accessed 2023-06-29

[55] Valente, G., Castellanos, A.L., Hausfeld, L., De Martino, F., Formisano, E.: Cross-validation and permutations in MVPA: Validity of permutation strategies and power of cross-validation schemes. NeuroImage

238, 118145 (2021). https://doi.org/10.1016/j.neuroimage.2021.118145. Accessed 2025-03-20

[56] Gwilliams, L., King, J.-R., Marantz, A., Poeppel, D.: Neural dynamics of phoneme sequences reveal position-invariant code for content and order. Nat Commun **13**(1), 6606 (2022). https://doi.org/10.1038/s41467-022-34326-1. Accessed 2024-05-30

[57] uxlfoundation/scikit-learn-intelex. Unified Acceleration (UXL) Foundation (2025). https://github.com/uxlfoundation/scikit-learn-intelex Accessed 2025-01-22

[58] Accou, B., Vanthornhout, J., Hamme, H.V., Francart, T.: Decoding of the speech envelope from EEG using the VLAAI deep neural network. Sci Rep **13**(1), 812 (2023). https://doi.org/10.1038/s41598-022-27332-2. Accessed 2023-06-12

[59] Pasley, B.N., David, S.V., Mesgarani, N., Flinker, A., Shamma, S.A., Crone, N.E., Knight, R.T., Chang, E.F.: Reconstructing Speech from Human Auditory Cortex. PLoS Biol **10**(1), 1001251 (2012). https://doi.org/10.1371/journal.pbio.1001251. Accessed 2025-03-20

[60] Maris, E., Oostenveld, R.: Nonparametric statistical testing of EEG- and MEG-data. Journal of Neuroscience Methods **164**(1), 177–190 (2007). https://doi.org/10.1016/j.jneumeth.2007.03.024. Accessed 2024-01-23

[61] Team, R.C.: R: A Language and Environment for Statistical Computing (2020). https://www.r-project.org/ Accessed 2023-06-29

[62] Bates, D., Mächler, M., Bolker, B., Walker, S.: Fitting Linear Mixed-Effects Models Using lme4. J. Stat. Soft. **67**(1) (2015). https://doi.org/10.18637/jss.v067.i01. Accessed 2023-04-12

[63] Lenth, R.V., Bolker, B., Buerkner, P., Giné-Vázquez, I., Herve, M., Jung, M., Love, J., Miguez, F., Riebl, H., Singmann, H.: emmeans: Estimated Marginal Means, aka Least-Squares Means (2023). https://cran.r-project.org/web/packages/emmeans/index.html Accessed 2023-06-29

[64] Barr, D.J., Levy, R., Scheepers, C., Tily, H.J.: Random effects structure for confirmatory hypothesis testing: Keep it maximal. Journal of Memory and Language **68**(3), 255–278 (2013). https://doi.org/10.1016/j.jml.2012.11.001. Accessed 2023-06-29

[65] Caucheteux, C., King, J.-R.: Brains and algorithms partially converge in natural language processing. Commun Biol **5**(1), 134 (2022). https://doi.org/10.1038/s42003-022-03036-1. Accessed 2025-02-12

[66] Mischler, G., Li, Y.A., Bickel, S., Mehta, A.D., Mesgarani, N.: Contextual feature extraction hierarchies converge in large language models and the brain. Nat Mach Intell **6**(12), 1467–1477 (2024). https://doi.org/10.1038/s42256-024-00925-4. Accessed 2025-02-14
