# Supplementary material for: Sensory sharpening and semantic prediction errors unify competing models of predictive processing in human speech comprehension
Source: PLoS Biol. 2026 Jan 9;24(1):e3003588. doi: 10.1371/journal.pbio.3003588 (PMC12788694; doi:10.1371/journal.pbio.3003588)
Supplement: Attachment — Submitted filename: HIB_response_r2.pdf [file pbio.3003588.s027.pdf]

Sensory sharpening and semantic prediction errors unify competing models of predictive processing in communication: Peer Review Round Two

Fabian Schneider and Helen Blank

*Corresponding author(s). E-mail(s): f.schneider@uke.de;
Contributing authors: helen.blank@rub.de;

1 General Response

We thank the anonymous reviewers for their valuable comments. To address them, we have reorganised parts of the manuscript and added further clarifications. As before, we mark original reviews in black, our responses in blue, and changes to the manuscript in green.

2 Response to reviewer #1

The authors have satisfactorily addressed our concerns and we think it will make an interesting contribution to the literature.

Thank you for your positive evaluation of the manuscript.

3 Response to reviewer #3

I thank the reviewers for their extensive efforts in responding to my previous comments (and those of the other reviewers). I found most of the responses very satisfactory and think the manuscript is much improved overall. However, I have to admit that I was not satisfied by the response to my first comment from the previous round of review. Specifically, in my reading, the manuscript still lacks a motivation for the use of the RSA approach. Again, I am not saying there is anything wrong with the approach, but no motivation

for using it is provided. The authors say in their response that they "explain why simply comparing morph sea-tea to the constituent words is inadequate". But I didn't see that explanation anywhere. Why is it inadequate? And why is the RSA not necessary when it comes to carrying out the analysis on semantic priors? For that analysis the RSA stage seems to be jettisoned and the analysis focuses on comparing the ability of different models to explain EEG data directly. This begs the question as to why one cannot do something similar for the sensory responses and just compare acoustic reconstructions directly? So again, the use of the RSA needs to be motivated with some text early in the main body of the manuscript so that readers can appreciate its importance. Otherwise, readers are likely to be unnecessarily confused or skeptical of the strength of the results or both.

Many thanks for your positive evaluation of our revision and for your comment. We now address 1) why RSA is employed for sensory content, 2) why a simple comparison of sea-tea is inadequate, and 3) why RSA is not employed for information theoretic measures. To address this more clearly, we also moved supplementary methods into the main manuscript:

> **Results 2.2, pp. 6, lines 124+**
>
> [...] To determine whether speaker-specific semantic priors influence low-level sensory representations early into the neural processing hierarchy, we employed an approach combining stimulus reconstruction models and within-item composite representational similarity analysis (cRSA). In this combined approach, stimulus reconstruction models allowed us to isolate sensory representational content (see Stimulus reconstruction of morphs), and cRSA enabled a comparison of the geometry of this sensory content jointly with predictors in different representational formats such as acoustics and semantics (see Modeling acoustic expectations as top-k predictions; Regressing composite representational similarity). [...]
>
> **Results 2.2, pp. 7, lines 173+**
>
> [...] Specifically, we selected the five most probable words under a speaker prior and combined them into one probability-weighted audio spectrogram (for analysis of the influence of the number of predicted words, see Supplementary Figures 3-4, Supplementary Tables 4-5). This was done because predictions in the brain are inherently distributional[1]: The brain cannot truly foresee the target word at each trial. Given the potential acoustic heterogeneity even for semantically related words, predictions should reflect the distribution of expected acoustic features instead of the target word exclusively (see Modeling acoustic expectations as top-k predictions). [...]
>
> **Results 2.3, pp. 11, lines 268+**

[...] Critically, modeling broadband EEG responses rather than decoded sensory representations allowed us to probe whether prediction errors still co-occurred at, for example, the acoustic or semantic levels as abstract information theoretic signals (i.e., surprisal) disjoint from sensory representations. Here, mTRF models were chosen in lieu of more complex cRSM regression because information theoretic measures are known to be directly detectable and are commonly probed in single-trial EEG signals[2,3]. [...]

Methods 4.9, pp. 26, lines 830+ (previously supplementary)

[...] Principally, a morph *sea-tea* given speaker context *nature* may be represented more like *sea* than *tea*–or vice versa. However, it is unlikely that the brain makes predictions that are this concrete, as it would require, for example, predicting *sea* at trial t (*sea-tea*|*nature*) but immediately thereafter *kayak* at trial $t+1$ (*kayak-cognac*|*nature*). In reality, the brain is assumed to make distributional predictions instead[1]. [...]

When modeling semantic feature predictions, this approach could be approximated by comparing a morph sea-*tea* to its constituent words because semantic contexts reflect strong categorical structure whereby any word in *nature* is more similar to other words of *nature* than other contexts. Critically, words that are similar in meaning can be highly heterogeneous in acoustic features and vice versa (e.g., *dog-canine* or *bear-bare*). Consequently, modeling acoustic expectations requires a more explicit commitment to distributional predictions.

References

[1] Friston, K.: The free-energy principle: a unified brain theory? Nat Rev Neurosci **11**(2), 127–138 (2010). https://doi.org/10.1038/nrn2787. Accessed 2024-01-23

[2] Heilbron, M., Chait, M.: Great Expectations: Is there Evidence for Predictive Coding in Auditory Cortex? Neuroscience **389**, 54–73 (2018). https://doi.org/10.1016/j.neuroscience.2017.07.061. Accessed 2022-10-11

[3] Donhauser, P.W., Baillet, S.: Two Distinct Neural Timescales for Predictive Speech Processing. Neuron **105**(2), 385–3939 (2020). https://doi.org/10.1016/j.neuron.2019.10.019. Accessed 2025-09-08
